# Supplementary material for: Experiences of menstruation in high income countries: A systematic review, qualitative evidence synthesis and comparison to low- and middle-income countries
Source: PLoS One. 2021 Jul 21;16(7):e0255001. doi: 10.1371/journal.pone.0255001 (PMC8294489; doi:10.1371/journal.pone.0255001)
Supplement: S1 Text — Identified via members of the Menstrual Health Hub (https://mhhub.org/community/global-mh-registry/) and partners of Menstrual Hygiene Day (https://menstrualhygieneday.org/get-involved/partnership/). Searched in September 2019 and updated in November 2020. (PDF) [file pone.0255001.s002.pdf]

S1 Text: Organisational and personal websites searched. Identified via members of the Menstrual Health Hub (https://mhhub.org/community/global-mh-registry/) and partners of Menstrual Hygiene Day (https://menstrualhygieneday.org/get-involved/partnership/). Searched in September 2019 and updated in November 2020.

"Monthlies" (Viafilm)
#HappyPeriod
28 Too Many
50 Cents. Period
Adventures in Menstruation
Advice to My Thirteen-Year-Old Self
Always
Asociación de Mujeres para la Salud
Aunt Flow
ayzh Inc
Be Girl
Be Prepared. Period
Beauty in Blood
Beppy Canada
Betty for Schools
big love sista
Binti International
Birmingham City University
Bloody Good Period
Body form
BodyLogic
Bright Girl Health
Brown Girl Magazine & The Desai Foundation
Brown University
Callaly
Capezio
Casco Bay Molding
Catapult
Celebrate Puberty Inc.
Celebration Day for Girls
Center for Endometriosis Care
Center For Erotic Intelligence
Central Australian Youth Link-Up Service
Cho Ngafor's Vocational Foundation
Círculo Perfeito
Claripharm
Clean Your Cup
Clue
CoMo
Confraria Vermelha
Conscious Period - www.consciousperiod.com
Coppetta Menstruale
Cora Women
Cotton Candy Eco Cloth
CouldYou
Crafts and Cramps
CrankyTown
Crown Abbey LLC
Cuptime
Cute Cotton
Cycle Seeds
CYCLO Menstruación Sostenible
Danish School of Education, Aarhus University
Dans Ma Culotte
Day of the Girl Summit
Days for Girls
Days for girls, Peebles and Thondwe Partnership
Diva International Inc.
Do It Now NOW
Earth Conscious
easy.
ECO-SER /Menstrupedia Comic Español
École polytechnique fédérale de Lausanne (EPFL) (Laura Symul)
Edinburgh Napier University
El Camino Rubí and Soy1Soy4
Emily Cherenack
Empowering Women. Period
End Poverty Now
EndoActive
ENDOMETRIOSIS ASSOCIATION
erdbeerwoche GmbH
Eva Teja
Evofem (SoftCup)
Face Of winter Netherlands
False Labels Global Inc
Feby Empowerment Inc.
Female Cup
FemCap, Inc.
FEMFLOW
Feminesse
Feminine Wear Ltd
FemmyCycle
Fertilidade Natural
Fields of Life
Fierce Coast Folks
Flying Inspiration
Freedom4girls
Fullfilling Destiny
Gashtrays and Venus Libido
Gifti
Girl Guiding
Girls Flow Free Girlsflowfree.org
Girls Globe
Girls Health Ed
Give Grace, Give Hope
Glad Rags
Global Communities
Global Health Initiative
Go With Your Flow
Grace & Green
Graduate Women international
Grand Challenges Canada
Guam Girl Scouts
Hands on Wellness / CycleBloom
Happeriod
Happy Cup
Harmonie Ženy - Jana Urbánková
Harvest Market Natural
Hello Flo
Helping Women Period
Hempact
Hey Girls UK
Hope and Dreams Initiative
Human Rights Watch www.hrw.org
I Support the Girls
I-Care
Institute for the Study of Human Rights, Columbia University
IntiMate Initiative
Irise International
Itwixie
Journalists for Human Rights
Ju Ju Menstrual Cup
Kærlig hilsen Underlivet
Keela
Kindara

Kliit Health

Kora Mikino

Korean Women's Environmental Network

La Source des Femmes

Ladybug for Girls Foundation, Inc.

Lara Owen

Let’s Talk Period Network (project by Brook and Plan International UK)

Lil-lets

Liverpool John Moores University Students Union

Liz Granger www.lizgranger.com

Lotus Liners

Love Cherish

Love Your Cycle – Helsinki

Love&Cherish

Luna Menstrual Health

Luna Pads

Lunar Circle

Lunaspongia

Lunette

Macheo

Marie Stopes International

Matronas Amane (Amane Midwives)

Maxim Hygiene Products

Medulla - wild cycles for wise women

Meloniesyre

Menkoppen

Mensen - forum för menstruation

Menskoppen

Menstrual Cup Co

Menstrual Health Hub Hive

Menstrual Hygiene Day Resources

Menstrual Matters

Menstruation Museum Amsterdam

Menstruation Research Network (and individual websites of members)

MenstruationMatters

MenstruationsNetzwerk

Mensual

MINT-E (menstrual information network teaching and empowerment project)

ModiBodi

Mondays - www.organicmondays.com

MonthlyCup AB www.menstrualcup.com

Moon + Womb

Mooncatcher

Mooncup

Moontime Harmony

Mother Nature Partnership

Moxie

My Best Period

My Puberty Party

My.flow

MYLILY

Nashiuti

Natracare www.natracare.com

Natural Flow

New Moon Pads

No More Taboo

Northwestern University Feinberg School of Medicine

NY Assembly

Odeeva

Ohnut Www.ohnut.co

One-Fertility

Ooshi GmbH

OrganiCup / Peace with the wold

PadBack

Party in My Pants

Penny Pack

Perfectly packaged you

PERIOD - The Menstrual Movement

Period Colouring Book

Period Equity

Period Packs, Inc.

Period Panteez

Period Partner

Period Positive

Period Talks

Period!

PHS Group Period Poverty Collective

PHSE Association

Plan Australia

Plan International

Plan UK

Plan Ireland

Poculum

POWER:Period.

Power2theWomb!

Precious Gems

Primal Wisdom

Project GIVE

Project Humanity

Project Pink Compassion

Red School

Red School Ltd

Rethinking Eve

Rose Temple Yoni Steaming

Rowe Clark H.S.

Ruby Cup by Ruby Life ltd.

Ruby May

Sara Lopez & Gush

Scensible Bags

Scottish Government

Share the Dignity

Shove it in your Vagina

Silvana Rigobon

Simply the Basics

Sister of Flow

Smart Health aka SuperIzzy

Social Good Moms

Society for Menstrual Cycle Research

Soy1Soy4.com

Sport England

State of Wi Legislature

Sustainable Cycles

Sustainable Sanitation Alliance

Sylvia's Sisters

Talking Periods

Tanya Dhingra

Tenda Vermelha

Terram Pacis

The Agenda

The Better Period

The Blobcast

The Butterfli Effect

The Circle

The Crimson Movement

The Cuntsultant

The Cup

The Cup Effect

The Female Company

The Flex Company

The Flow Menstrual Cycle App

The Healthy Womb

The intiMate Initiative UG (haftungsbeschraenkt)

The Kota Alliance

The Monthly: a project of DC Diaper Bank

The Moon in Our Blood

The Period Project

The Period Purse

The Period Store

The Real Period Project

The Red Box Project

The Red Elephant

The Red Web

The Red Web Foundation

The University of Queensland

The Waratah Project

Thieut Inc.

Thinx

This is a tampon - instagram handle

Time of the Month (TOTM)

Top Organic

Transparent Heart

Tsuno

Two Rags

UhuruPads

UK government’s period poverty task force

UN Women

UNFPA

UNICEF

Unite Youth Dublin Activist Group

University of Illinois at Chicago

University of Leeds

Untabooed

Valley Electronics AG (Daysy)

VrouwenBron

Wā Collective

WASH Canada

WASH United

WaterAid

We-Stap

Woman Care Global

Women and Girls

Women For Independence

Women in Sanitation and Health "WiSH"

Women on Wings

Women's Environmental Network (WEN)

Women's Global Health Innovations

WoMena Denmark

WonderCup

World Menstrual Network

World Pulse

Yoga Buds, Yoga Blossoms, Yoga Flowers

Yoni

Young Jewels Foundation

Your Sacred Cycle

Zyklusachtsamkeit
