# Supplementary material for: Experiences of menstruation in high income countries: A systematic review, qualitative evidence synthesis and comparison to low- and middle-income countries
Source: PLoS One. 2021 Jul 21;16(7):e0255001. doi: 10.1371/journal.pone.0255001 (PMC8294489; doi:10.1371/journal.pone.0255001)

S2 Text: Menstrual health researchers contacted directly (October 2019 and November 2020)

Milena Bacalja

Bettina Bildhauer

Christina Bobel

Chris Bonell

Liita N. Cairney

Marisa Carnesky

Ruth Cochrane

Sara De Benedictis

Nina Hall

Celia Hodson

Bee Hughes

Tania John

Ina Jurga

Danielle Keiser

Sally King

Victoria Newton

Lara Owen

Penny Phillips-Howard

Carrie Purcell

Kate Rahnejat

Emma Ross

Camilla Mørk Røstvik

Kate Sang

Kelly Shephard

Marni Sommer

Kay Standing

Emily Stewart

Jane Ussher

Rachel Vette

Katy Vincent

Shailini Vora

Pamela Warner

Annalise Weckesser

Helen Weiss

Gemma Williams

Inga Winkler

Sarah Zipp

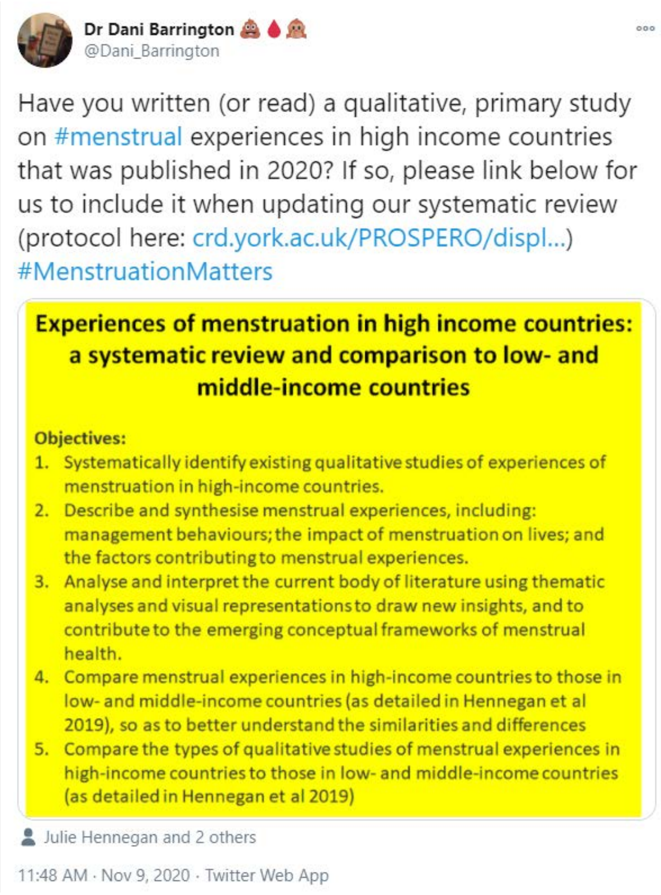

Supplement: S2 Text — (PDF) [file pone.0255001.s003.pdf]
