# Supplementary material for: Experiences of menstruation in high income countries: A systematic review, qualitative evidence synthesis and comparison to low- and middle-income countries
Source: PLoS One. 2021 Jul 21;16(7):e0255001. doi: 10.1371/journal.pone.0255001 (PMC8294489; doi:10.1371/journal.pone.0255001)
Supplement: S1 Table — References in black boxes indicate they are from a study with multiple publications included in this review (publications from the same study are grouped together in Table 2). Quality is assessed at the publication level here and at the study level for analysis. Adapted from the EPPI-Centre Checklist detailed in Rees, R., Oliver, K., Woodman, J., Thomas, J. (2009) Children’s views about obesity, body size, shape and weight: A systematic review. EPPI-Centre, London:UK [18]. (PDF) [file pone.0255001.s004.pdf]

S1 Table: Quality appraisal of included studies. References in black boxes indicate they are from a study with multiple publications included in this review (publications from the same study are grouped together in Table 2). Quality is assessed at the publication level here and at the study level for analysis. Adapted from the EPPI-Centre Checklist detailed in Rees, R., Oliver, K., Woodman, J., Thomas, J. (2009) *Children's views about obesity, body size, shape and weight: A systematic review*. EPPI-Centre, London:UK [1].

Colour Key	1. Were steps taken to increase rigour in the sampling?	2. Were steps taken to increase rigour in the data collected?	3. Were steps taken to increase rigour in the analysis of the data?	4. Were the findings of the study grounded in/supported by the data?	5. Please rate the findings of the study in terms of their breadth and depth	6. To what extent does the study privilege the perspectives and experiences of those who menstruate?
(green)	Yes, a fairly thorough attempt was made	Yes, a fairly thorough attempt was made	Yes, a fairly thorough attempt was made	Good grounding/support	Good/fair breadth and depth	A lot
(yellow)	Yes, several steps were taken	Yes, several steps were taken	Yes, several steps were taken	Fair grounding support	Good/fair depth but very little breadth OR Good/fair breadth but very little depth	Somewhat
(orange)	Yes, a few steps were taken	Yes, a few steps were taken	Yes, a few steps were taken			A little
(blue)	No, not at all/not stated/can't tell	No, not at all/not stated/can't tell	No, not at all/not stated/can't tell	Limited grounding/support	Limited breadth or depth	Not at all
	**Overall trustworthiness, consider questions 1 to 4**				**Overall usefulness/relevance, consider the review question, whether participants were from a specific sub-population of those who menstruate in HICs (e.g. experience endometriosis, only those who recently reached menarche, intellectually disabled), and questions 5 and 6**	
	High				High	
	Medium				Medium	
	Low				Low	

Reference ID	1. Rigor in sampling	2.Rigor in data collection	3.Rigor in analysis	4.Findings supported by the data	5.Breadth and depth of findings	6.Privileges perspectives of those who menstruate
Adams-Matthews 2009 [2]	Very small, convenience sample (4 women in groups known to author).	A lot of effort to make sure participants were at ease, and lots of methods used to triangulate findings.	Very thorough description of analysis. Findings were reflexive and checked with the interviewee.	Very detailed narratives about the experiences, and interpretations of them, for each participant.	Very deep consideration of issues of each participant. But difficult to know breadth as sample was so small.	There seems to have been a lot of effort made to put participants at ease and capture all the topics they wanted to discuss, as well as those necessary to the research question.
	Trustworthiness: Medium				Relevance: Low (very small, convenience sample)	
Allen & Goldberg 2009 [3]	Mostly white and all educated Students in course on human sexuality, may be more open to "taboo" sexual practices? "The goal of the course was to explore the diversity of human sexuality using global perspectives from interdisciplinary sources including biological, historical, developmental, psychological, and sociological approaches".	Private narratives were a good way of getting women to feel comfortable opening up (may not have done so via interviews). Piloted in first year and when it worked, continued for years 2 and 3.	Memos + constant comparative method of coding, multiple coders with refinement until 100% agreement. Diversity in perspective explored.	Pseudonymised quotes provided demonstrating multiple perspectives.	Topic deeply explored over a range of different perspectives.	Menstruators own voices at the forefront, method allowed for intimate sharing without discomfort.
	Trustworthiness: High				Relevance: High	

Reference ID	1. Rigor in sampling	2.Rigor in data collection	3.Rigor in analysis	4.Findings supported by the data	5.Breadth and depth of findings	6.Privileges perspectives of those who menstruate
Allyn et al. 2020 [4]	Sample recruited mostly through emailing university students, then purposively selecting respondents with menstrual pain rated 6 or above. Volunteers were excluded if they were taking hormonal contraceptives.	Unclear whether the semi-structured interview tool was piloted. The tool itself not provided. Processes for consent and assent detailed.	The data analysis method was systematic.	Many pseudonymised quotes support findings.	Perspectives of multiple participants considered and contrasted.	The coding framework was inductive, and from the quotes provided it appears questions were appropriately open-ended. Unclear who designed the research.
	Trustworthiness: High				Relevance: High<br>Focus of study was on the direct experience of those who menstruate. Primary dysmenorrhea sufferers are a sub-group, but a large enough sub-group to be of widespread importance	
Amann-Gainotti 1986 [5]	Method is very short, it seems they were a "random" selection, but not sure how they were recruited.	Very little detail.	Only basic statistics are obvious, nothing on qualitative analysis.	Multiple quotes support the findings, with age of respondent noted.	Describes answers to questionnaire with limited analysis.	General population of school-aged participants. Unclear how consent was obtained, participants put at ease, or how analysis was conducted.
	Trustworthiness: Low				Relevance: Low	
Andrews 1985 [6]	Relatively small sample, only 13 girls were pre-menarcheal, some information given about living situations, etc.	Parental consent taken, interviews conducted in private, interview style allowed for probing and tailoring questions.	A lot of inferring in the results without necessarily being backed up by data or citations.	Some illustrative quotes provided, but without attribution.	A range of issues but across a restricted sample of post-menarcheal girls.	General topics were deductive, but opinions/experiences were deductive. Girls interviewed in private.
	Trustworthiness: Medium				Relevance: Medium	
APS Group Scotland 2018 [7]	Mostly convenience sampling of recipients of intervention.	Lots of triangulation to collect multiple perspectives through different methods.	Qualitative data analysis was deductive and completed by two researchers, with triangulation to investigate findings.	Some quotes given; majority of the results presented are quantitative.	Qualitative data collection limited compared to quantitative, qualitative data itself was limited in depth. Mostly about the intervention rather than experiences of interest.	Participants all menstruate but difficult to discern how the research was designed. Efforts were made to put participants at ease and ethical practices were considered/followed.
	Trustworthiness: Medium				Relevance: Medium	
Armeni 1997 [8]	Researcher claims that the sampling was random, but no details given of how participants were recruited. The choice of two cohorts of women makes sense in evaluating changes in menstrual experiences across generations. Advertisements for menstrual products during Twentieth Century also analysed.	The data collection instrument is provided, but it is unclear whether it was piloted. However, the author did use a reflexive approach to the interviews, such that most became "unstructured conversations". The author visited women twice, aiming to build rapport in the first interview, and went to lengths to ensure participants were put at ease.	It is unclear how the author used oral history methods to analyse the data herself, although analysis with and by the participants during interviews was undertaken. The author explored the opinions of deviants in the sample, allowing for more diversity to be included. One participant from each cohort was involved in critiquing the author's thesis. Difficult to discern whether the analysis was guided by preconceptions.	Named quotes from participants or advertisements given in most cases.	In depth exploration of several women's menstrual experiences over the lifespan.	The author did use a reflexive approach to the interviews, such that most became "unstructured conversations". The author visited women twice, aiming to build rapport in the first interview, and went to lengths to ensure participants were put at ease. Analysis with and by the participants during interviews was undertaken. The author considers her own positionality throughout.
	Trustworthiness: Medium				Relevance: High	

Reference ID	1. Rigor in sampling	2.Rigor in data collection	3.Rigor in analysis	4.Findings supported by the data	5.Breadth and depth of findings	6.Privileges perspectives of those who menstruate
Armour et al. 2016 [9]	Purposive sampling from results of quantitative survey, of women enrolled in RCT investigating acupuncture and menstrual symptoms.	Conditions of ethical approval followed, only single interview, those who had provided medical advice did not interview participants.	Theoretical sampling with constant comparative analysis. Multiple researchers involved in analysis.	Ample pseudonymised quotes provided to support findings.	Good breadth and depth, although focus is on experience of RCT, not menstruation.	Steps taken to put participants at ease through use of Skype for interviews. Some consideration of positionality.
	Trustworthiness: High				Relevance: Medium	
Armour 2015 [10]	Women enrolled in RCT investigating acupuncture and menstrual symptoms.	Conditions of ethical approval followed, only single interview, those who had provided medical advice did not interview participants.	Theoretical sampling with constant comparative analysis. Multiple researchers involved in analysis.	Ample pseudonymised quotes provided to support findings.	Good breadth and depth, although focus is on experience of RCT, not menstruation.	Steps taken to put participants at ease through use of Skype for interviews. Some consideration of positionality.
	Trustworthiness: High				Relevance: Medium	
Artschwager 1981 [11]	Purposive (although sounds more convenience) sample of Mexican American women from a family planning clinic and going door to door.	Can't tell.	Can't tell.	Can't tell.	Multiple responses presented/explored, but not in depth.	Difficult to tell.
	Trustworthiness: Low				Relevance: Low	
Beausang & Razor 2000 [12]	Study participants were all enrolled in a community college human sexuality course and the assigned topic of the narrative was sexuality, not menstruation specifically. Re-analysed later to investigate menarche as it was commonly mentioned.	Students were able to write whatever they felt was relevant and did not have to write about topics which made them uncomfortable. Consent was obtained, but consent forms were kept separate from essays.	Unclear how systematic data analysis was.	Some demonstrative quotes provided.	Fair breadth, difficulty assessing depth of analysis.	Essay topic allowed for menstruators to write about experiences that were important to them.
	Trustworthiness: Medium				Relevance: Low<br>Specific focus on menarcheal education	
Bishop 1999 [13]	Snowball sampling and recruitment via various appropriate networks.	Steps were taken to put participants at ease through a thorough method of confidential recruitment and return of questionnaires. Some of the quantitative scales have been validated previously; there did not seem to be any specific validation for this study.	Data analysis methods were systematic.	Qualitative data support the findings	Limited conceptual development.	Confidentiality steps were in place. There was an opportunity for open-ended responses, which could be coded and compared to quantitative responses.
	Trustworthiness: Medium				Relevance: Low	
Bobier 2020 [14]	Small sample from snowball recruitment, reached saturation. Recognised weakness of friends recruiting friends.	Unclear whether the tool was piloted, but it was reflexive to allow participants to share in a natural way. Researcher made efforts to put participants at ease and required both parental consent and participant assent to take part in the study.	Very limited detail, just says interviews were 'coded inductively'	The data presented do support the findings, although the small number of examples somewhat limits generalisability.	Fair depth and linkage of results to interpretations, limited topics.	Interviews were reflexive and allowed participants to express themselves on their own terms. The positionality of the researcher was considered. Analysis unclear except that it was inductive.
	Trustworthiness: Medium				Relevance: Medium	

Reference ID	1. Rigor in sampling	2.Rigor in data collection	3.Rigor in analysis	4.Findings supported by the data	5.Breadth and depth of findings	6.Privileges perspectives of those who menstruate
Botello-Hermosa & Casado-Mejia 2015 [15]	Sampled 4 individuals (who currently or previously menstruated) within 6 different age groups. Mixture of rural and urban, and education levels. Unclear how the individuals were recruited.	Informed consent taken, but unclear on the content of semi-structured interviews.	Data analysis was systematic and balanced, although was very prescriptive (guided by pre-conceptions).	Multiple quotes given to support most findings, identified by age and location.	Fair breadth but very little depth into topics.	Voices of menstruators privileged, but difficult to understand how semi-structured data collection methods were developed.
	Trustworthiness: Medium				Relevance: Medium	
Bransen 1992 [16]	Author wasn't aiming for representative sample, just wanted to "find my way into the phenomenon".	Can't tell.	Can't tell.	Quotes provided for findings, but not numbered/pseudonymised.	Fair depth but limited breadth in analysing the data.	Study privileges the perspectives of those who menstruate, but difficult to understand how the methodology was developed.
	Trustworthiness: Low				Relevance: Low	
Brantelid 2014 [17]	Snowball sampling.	Data collection tools were piloted and amended.	Systematic.	Quotes provided with pseudonyms.	Good depth and breadth.	Voices of menstruators privileged.
	Trustworthiness: High				Relevance: Medium	
Briggs 2020 [18]	Unclear how participants were recruited but was a purposive sample appropriate to early investigations of a sensitive topic. Some demographics provided.	Overall topics of interview tool provided, but not the instrument itself, and unclear whether it was piloted. Tool may have been flexible and comprehensive, but it is unclear. Several steps were taken to obtain informed consent and reduce power differentials between the researcher and participants.	The data analysis method was systematic but was performed by a single researcher and did not involve sharing of results (during analysis) with participants.	Some pseudonymised quotes provided to support findings.	Little transformation of data, some quotes provided but mostly backed up by existing literature. Some range of issues considered.	Analysis was inductive and the researcher considered her positionality in conducting data collection and analysis. Questions appear to be open-ended but unclear without a copy of the instrument.
	Trustworthiness: Medium				Relevance: Medium	
Britton 1996 [19]	Sampling wasn't meant to be representative, but it's also unclear how it was recruited.	No details given on the method beyond it being a semi-structured interview.	No method of analysis stated.	Pseudonyms used for quotes, but no details given of which pseudonyms identify with each country background. Not always clear which details are findings from primary data or supporting evidence from previous publications.	Looks at multiple topics from multiple viewpoints but does not go into the experiences of individual women in depth; is quite surface level.	Quotes are clearly from women who menstruate, but without details of the method, method development or analytical techniques, it's impossible to know more than that.
	Trustworthiness: Low				Relevance: Medium	
Brookes & Tennant 1998 [20]	Can't tell.	Can't tell.	Can't tell.	Limited grounding/support.	Fair breadth but limited depth to individual stories.	To some extent.
	Trustworthiness: Low				Relevance: Medium	
Brown et al 2020 [21]	Purposive sampling of elite athletes. Demographics provided and use of hormonal contraceptives (and their impact on menstrual experiences) investigated.	The data collection tool was piloted several times before the final iteration. Efforts were made to put participants at ease through the choice of interviewer and her background.	Data analysis was thorough and multiple authors were involved. Positionality was considered.	Good grounding with numbered codes provided for findings.	Good/fair breadth and depth.	Several steps taken to develop instrument with menstruators and make participants comfortable. Ethical approval was granted and followed.
	Trustworthiness: High				Relevance: High	

Reference ID	1. Rigor in sampling	2.Rigor in data collection	3.Rigor in analysis	4.Findings supported by the data	5.Breadth and depth of findings	6.Privileges perspectives of those who menstruate
Bullo & Hearn 2020 [22]	Purposive sampling for women with endometriosis via social media. Limited demographic data beyond age.	Participants given freedom to lead interview. Unclear whether the method was piloted.	Very detailed and systematic analytical procedure.	Pseudonymised quotes supported findings.	Focused almost exclusively on the metaphors used to express pain.	Participants drove the interview topics to be those of most importance to them. Analysis was inductive. Positionality of researchers considered throughout.
	Trustworthiness: High				Relevance: Medium<br>Mostly focused on the metaphors used and only with women who experience endometriosis.	
Burbeck 2014 [23]	Very small sample size, snowball sampling. But did state that they excluded anyone using oral contraceptives from sample.	Interview piloted and amended, only one interviewer in the room.	Details of analytical approach given, including re-analysis over multiple readings.	Quotes identified by pseudonyms, enough to support findings.	Range of issues and depth in exploring individual experiences.	Appropriate methods in place for protection of confidentiality and comfort of participants, and analysis was iterative drawing on findings.
	Trustworthiness: High				Relevance: High	
Burrows & Johnson 2005 [24]	Convenience sampling for adolescent girls willing to speak on topic (and where gatekeeper permission was granted).	Unclear, although some attempt to make participants feel comfortable.	Analysis was systematic, but no codebook, etc., provided.	Quotes with pseudonyms provided to support findings. Some demographic details provided.	Fair breadth and depth.	Efforts to make participants comfortable and discuss issues of concern during FGDs.
	Trustworthiness: Medium				Relevance: Medium	
Byles et al. 1997 [25]	Participants were randomly recruited. Demographic details of individual interviewees not provided.	Semi-structured focus group discussions. Unclear whether piloted. Example questions provided.	Appears to be systematic, but limited details provided.	Quotes provided to support findings, but not pseudonymised.	Breadth of findings but individual experiences not investigated in depth.	Unclear how method was developed. Measures were taken to ensure women were comfortable.
	Trustworthiness: Medium				Relevance: Medium	
Cattaneo 2000 [26]	Sample was all women the researcher knew through her networks. But she is clear that her work is about families of women in this particular area, so the sampling method may be justified on that.	Informed consent processes were followed, and great effort was taken to put participants at ease. It's unclear whether the interview instrument was piloted, but the life histories did take place after the interviews, so the researcher was by then comfortable that it was appropriate.	The analytical method unclear, but the author states that this is due to a desire to keep the women as subjects rather than "objects to be studied". Mostly, the author is just re-telling the women's stories, summarising them, and backing them up with literature.	The summaries of the similarities and differences in experiences are all supported by detailed, named quotes.	Very deep investigation of the experiences of individual women, and consideration of the antecedents of these, supported by the literature. A small study population, but the generational breadth adds great value.	The researcher thoroughly considered her positionality as a member of the same society as participants, and as a menstruating woman herself. Where possible, she asked families who provided life histories to review and approve them before publication.
	Trustworthiness: Medium				Relevance: High	
Chapple 1999 [27]	Purposive sampling for women who had experienced/reported menorrhagia (may or may not have consulted about it), specifically to over-represent South Asian women. Characteristics of individual participants not provided.	Interview questions not clear	Analysis reported as being systematic, but limited details given.	Pseudonymised quotes support findings.	Findings were broad but not deeply investigated.	Menstruators not obviously involved in method development. Details of ethical consideration/approval not given.
	Trustworthiness: Medium				Relevance: Medium	

Reference ID	1. Rigor in sampling	2.Rigor in data collection	3.Rigor in analysis	4.Findings supported by the data	5.Breadth and depth of findings	6.Privileges perspectives of those who menstruate
Chen et al 2018 [28]	Purposively selected from larger study using screening questionnaire. Demographic details of sample provided, but not of individual participants.	Only one qualitative question given, detailed in paper.	Analysis was systematic, but details of codes, etc, not provided.	Findings supported by data but quotes not numbered/pseudonymised.	Broad and deep findings presented.	Menstruators who experience dysmenorrhea not obviously involved in method development. Details of ethical considerations discussed.
	Trustworthiness: High				Relevance: Medium	
Chou et al. 2008 [29]	Purposive sample from three institutions. Details of individual participants not provided.	Topics of open-ended questionnaire presented. Unclear whether it was piloted.	Analysis was systematic and details of coded themes provided.	Pseudonymised quotes provided to support findings.	Broad and deep discussion of various facets of menstruation.	One of few studies to privilege the voices of women with intellectual disabilities rather than a parent/caregiver. Measures in place to make participants feel at ease.
	Trustworthiness: High				Relevance: Medium<br>Sub-population of women with an intellectual disability	
Chrisler et al 2016 [30]	Obviously not a statistical sample, but the first study on this topic so they were going as wide as possible. Group would have been self-selecting.	Majority of the data collection was quantitative, which is well designed. Some details given of open-ended (qualitative) questions. Unclear how this was developed.	No details provided on analysis of qualitative data	Quotes provided but without pseudonyms or demographic characteristics.	Limited breadth/depth for qualitative findings.	Not clear that all conclusions were drawn from those who menstruate.
	Trustworthiness: Medium				Relevance: Low	
Christoforou 2018 [31]	Snowball, but then purposive to get range of views.	Interview guide was piloted, and reflexivity practiced throughout data collection.	Analysis was systematic, but no codebook, etc., provided.	Lots of referring to literature in the results section, and sometimes doing so with examples that don't quite fit.	Fair breadth, limited depth.	Measures were taken to ensure comfort of interviewees and pilot interviewees informed method development.
	Trustworthiness: Medium				Relevance: High	
Clark 2012 [32]	13 women recruited via the national Endometriosis Charity. There does seem to be some snowball sampling within the 13. Recruiting via Endometriosis UK was appropriate.	Informed consent processes were followed, and the researcher tried to make participants comfortable. The data collection instrument is included, although it is unclear whether it was piloted.	Data analysis methods were systematic. Researcher investigated a positive deviant example.	Many numbered quotes provided to support findings.	Range of issues explored in depth.	Researcher is a woman with endometriosis who also thoroughly considers her positionality when conducting analysis.
	Trustworthiness: High				Relevance: Medium	
Cooper & Koch 2007 [33]	Recruitment was initially via a clinic, so may have recruited women with more health concerns (as noted by authors). When this recruitment strategy didn't work, women were recruited via snowball sampling. Limited demographics on participants beyond them identifying as African American and the age given for individual quotes.	The data instrument is unavailable but can be inferred that it was very reflexive (as women often wanted to talk more about various topics, or had follow-up questions, and these were allowed). Unclear whether it was piloted. Informed consent procedures were followed. There was no follow-up.	The data analysis method was systematic, but the paper doesn't present diverse perspectives or search for alternative meanings.	The quotes are numbered individually, and generally support the findings, but there may be some over-interpretation of data.	Limited comparison of experiences between women, and it isn't clear from individual quotes whether they adequately support author inferences.	Grounded theory was used, but there was no follow-up, and it is not clear that African American women were involved in designing the research.
	Trustworthiness: Low				Relevance: Low	

Reference ID	1. Rigor in sampling	2.Rigor in data collection	3.Rigor in analysis	4.Findings supported by the data	5.Breadth and depth of findings	6.Privileges perspectives of those who menstruate
Costos et al. 2002 [34]	Seems that each student chose someone in their life to interview – a novel method.	Informed consent, and students would have interviewed people they felt comfortable with, but there's no way of telling how data collection really happened across the study. However, results may have been better quality because of this interviewer/interviewee relationship.	Analysis seems to have been systematic and involved 3 individuals. Diversity of opinions were explored. There was no way of feeding back results to participants for triangulation.	Lots of quotes provided, but not pseudonymised.	In depths answers given by participants, but not discussed in depth by authors.	Difficult to discern as authors did not interview participants. Likely menstruators' voices were privileged by the sampling technique/comfort with interviewers.
	Trustworthiness: Medium				Relevance: Medium	
Deforest 2007 [35]	Author seeks diversity of ethnicities, culture, etc., but it seems unlikely that with a sample size of seven there could be any generalisable findings to specific ethnicities/cultures. All women recruited were known to the researcher or her acquaintances. Some demographic details of each participant given. By contrast, collection of secondary literature for analysis seems to have been methodical.	The data collection tool was developed for a face-to-face interview but ended up being conducted via email exchanges. It was not piloted (although the questions did come from a previous study, Lee 2002). Participants received and signed information and consent forms. Each individual was later interviewed over the phone.	Data analysis was systematic, but presentation of findings from the interviews is mostly a summary and includes personal opinions of the research not backed up with quotes or citations.	Presentation of findings from the interviews is mostly a summary and includes personal opinions of the researcher not supported by quotes or citations.	Small sample of women and data is summarised rather than analysed.	Difficult to discern whether the researcher considers her own positionality, given that she voices her own opinion throughout the summaries/analysis.
	Trustworthiness: Low				Relevance: Medium	
DeMaria et al. 2019 [36]	Did involve snowball sampling, but also widely advertised aiming for representative sample.	Built rapport so women would be comfortable, and interview tool was reflexive even within interviews. Tool not provided.	Analysis was systematic and explored diversity in perspective.	Findings supported by quotes with key characteristics, but not pseudonyms.	Broad findings but explored in depth.	Unclear who was involved in developing data collection tool, but it was reflexive to participant needs.
	Trustworthiness: High				Relevance: Medium	
DeMaria et al. 2019 [37]	Convenience and snowball sampling via social media. Diversity likely impacted by requirement to be fluent in conversational English. Demographics (age, education, sexuality) provided.	The data collection tool is provided, but it is unclear whether it was piloted. Steps were taken to make participants comfortable and ethical consent procedures were followed.	Multiple researchers involved in systematic analysis/interpretation. Results were not fed back to participants for confirmation.	Quotes provided, but without numbers/pseudonyms.	Limited breadth and depth specifically related to menstruation	Responses were open-ended and used probing. Unclear who designed the research. Some positionality considered. Consent procedures followed.
	Trustworthiness: Medium				Relevance: Low<br>More generally about genital self-image, with sub-component on menstruation	
Denny et al. 2011 [38]	Mostly snowball sampling, but for the purposes of this study, I think it sufficed - were looking for general cultural trends. Characteristics of participants provided.	Lots of thought given to development of topic guide and how they were most likely to get in depth answers. FGD guide provided.	Lots of people reviewed the data, including community facilitators.	Quotes given with ethnicity, but not indicative of individuals within ethnic group.	Broad findings but generally not investigated in depth.	Community facilitators were recruited from target minority ethnic groups.
	Trustworthiness: High				Relevance: High	

Reference ID	1. Rigor in sampling	2.Rigor in data collection	3.Rigor in analysis	4.Findings supported by the data	5.Breadth and depth of findings	6.Privileges perspectives of those who menstruate
Dillaway et al. 2013 [39]	Had trouble finding the sampling strategy of the original programme as the name of programme is given, but no citations. However, participant selectin/recruitment appears sound for the research question and demographic characteristics are given.	Unclear whether interview tool was piloted but was very reflexive to gathering experiences and opinions of participants.	Very thorough description of systematic coding process.	Many quotes provided with pseudonyms and key characteristics of participants.	Broad and deep findings.	Many measures were in place to ensure women with spinal cord injuries were made to feel comfortable and to express their opinions. Unclear whether they were involved in methodology development.
	Trustworthiness: High				Relevance: Medium<br>Sub-population of women with spinal cord injuries	
Ditchfield & Burns 2004 [40]	Purposive sample for women with learning disabilities. Characteristics of participants provided.	Unclear whether interview tools were piloted or what questions were asked. However, care was taken to obtain informed consent and put participants at ease.	Little details of how thematic analysis was conducted (e.g. no codebook provided)	Many quotes given but often not pseudonymised, so it is unclear whether certain individuals were over-represented.	Small sample but investigated multiple topics, e.g. identity, preparedness, impacts, attitudes.	Unclear who was involved in developing the study, but one of few about menstruation and learning disabilities where the voice of the menstruator is privileged above that of the parent/caregiver.
	Trustworthiness: Medium				Relevance: Medium<br>Sub-population of women with learning disabilities.	
Donmall 2013 [41]	Very small sample - 6 - recruited through friends and family.	Can't tell.	Analytical method thoroughly described and systematic.	Results supported by many pseudonymised quotes. Demographic details do not accompany the pseudonyms.	Focus on experiences at menarche.	Unclear who was involved in developing interview tool or what it entailed (with regards open-ended questions, etc.). Unclear what consent procedures, etc., were followed.
	Trustworthiness: Low				Relevance: Medium	
Elson 2002 [42]	Recruited via word of mouth and medical referrals, did have to have had a non-cancerous surgery that has required hysterectomy.	Can't tell.	Yes, looked at diversity of opinions, e.g. father's involvement, and how that is different from the literature.	Results supported by many pseudonymised quotes. Demographic details do not accompany the pseudonyms.	A range of issues are explored, with depth of individual experiences.	Only those who formerly menstruated interviewed.
	Trustworthiness: Medium				Relevance: Medium	
Eriksen 2016 [43]	At study level, recruited via Autism databases through the Interactive Autism Network. For this component, purposive sampling undertaken to ensure heterogeneity across age at menarche, ASD diagnoses, age, and race and ethnicity. Demographic details provided for each participant.	Informed consent and assent procedures followed. Interview format chosen by parent-daughter dyad. The data collection instrument was developed with input from mentors and experts in the field. Early interviews were reviewed, and the guide was adapted and supplemented as topics emerged.	Analysis was systematic and data collection and analysis continued until saturation was reached. Multiple researchers involved in developing codes and actual coding. Findings do not appear to have been influenced by pre-conceptions, on the contrary, there is very little reference to existing literature to explain the findings.	Quotes supported findings, but there is little use of existing literature to interpret the findings in a wider sense (i.e. beyond the narratives of the dyads).	Good/fair breadth and depth when considering the responses/findings provided by mothers.	Mothers and daughters interviewed, but more weight given to mothers' stories.
	Trustworthiness: Medium<br>The study is well designed, but it is worrying that there is so little interpretation of the data when compared to other studies - e.g. puberty more generally or ASD more generally				Relevance: Medium<br>Sub-population	

Reference ID	1. Rigor in sampling	2.Rigor in data collection	3.Rigor in analysis	4.Findings supported by the data	5.Breadth and depth of findings	6.Privileges perspectives of those who menstruate
Fahs 2011 [44]	Participants recruited via advertisements, then purposively selected. Appropriate to sensitive topic but not necessarily representative.	Unclear whether tools were piloted. Interviews were open-ended and flexible where useful.	Data analysis appears to be systematic and explored varying perspectives.	Findings supported by pseudonymised quotes which also indicated the participants' age, race and sexual identity.	Presents broad findings of sexual experience of women of varying demographics.	Only those who menstruate interviewed. Unclear who was involved in designing the data collection instrument.
	Trustworthiness: High				Relevance: High	
Fahs 2014 [45]	Participants recruited via advertisements, then purposively selected. Appropriate to sensitive topic but not necessarily representative.	Unclear whether tools were piloted. Interviews were open-ended and flexible where useful.	Data analysis appears to be systematic and explored varying perspectives.	Findings supported by pseudonymised quotes which also indicated the participants' age, race and sexual identity.	Presents broad findings of sexual experience of women of varying demographics.	Only those who menstruate interviewed. Unclear who was involved in designing the data collection instrument.
	Trustworthiness: High				Relevance: High	
Fahs 2020 [46]	Participants recruited via advertisements, then purposively selected. Appropriate to sensitive topic but not necessarily representative.	Unclear whether tools were piloted. Interviews were open-ended and flexible where useful.	Data analysis appears to be systematic and explored varying perspectives.	Findings supported by pseudonymised quotes which also indicated the participants' age, race and sexual identity.	Presents findings specific to menstrual experiences.	Only those who menstruate interviewed. Unclear who was involved in designing the data collection instrument.
	Trustworthiness: High				Relevance: High	
Fernández-Martínez et al. 2020 [47]	Purposive sampling of nursing students. All were invited to participate and then selected based on criteria (mainly experiences of PD). Did not distinguish responses of those taking hormonal contraceptives.	Data collection instrument is provided and allowed for probing. Researchers attempted to address power relations in the way they facilitated via Zoom. It is unclear whether data collection tools were piloted.	Data analysis was systematic, considered various quality aspects, and was performed by three researchers. All themes detailed in Supplementary Material.	Limited quotes are given in the transcript, but they are detailed in the Supplementary Material, where quotes are numbered to individual FGDs (not participants).	Fair depth but difficult to know how many individuals in each focus group provided responses.	The FGD setting allowed for probing, although it also may have been somewhat stilted using Zoom/having to electronically raise hands to participate. The positionality of the researchers was considered from the beginning.
	Trustworthiness: High				Relevance: High	
Findlay et al. 2020 [48]	Sampling was purposive, which makes sense for the research question, but it is unclear how recruitment was undertaken. Some characteristics provided, including hormonal contraceptive use.	Interview topics provided, but unclear whether they were piloted. Unclear where interviews were conducted and whether efforts were made to put participants at ease.	Analysis was systematic and a codebook provided. Experiences between individuals were contrasted.	Numbered quotes support all findings.	Good/fair breadth and depth.	Questions were open-ended and analysis was inductive. Ethics approval was granted, but unclear how menstruators were put at ease.
	Trustworthiness: High				Relevance: High	
Fingerson 2005 [49]	Unclear on sample selection and recruitment.	Tools were piloted, male co-researcher engaged to run focus groups and interviews with boys. Does not seem to have been follow-up of findings but did triangulate using FGDs/individual interviews.	Analytical method described, some diversity of perspective explored, particularly through separate gender focus groups.	Findings supported by pseudonymised quotes, including age.	Several topics explored deeply.	Spoke to both boys and girls, but as the focus was on menstrual discourse (not experience) this was important. Unclear whether students/parents had to provide consent/assent to participate. Did try to put participants at ease, especially with gender-matching FGDs and interviews with facilitators.
	Trustworthiness: Medium				Relevance: Medium<br>Focus is more about menstrual discourse than actual experience	

Reference ID	1. Rigor in sampling	2.Rigor in data collection	3.Rigor in analysis	4.Findings supported by the data	5.Breadth and depth of findings	6.Privileges perspectives of those who menstruate
Fingerson 2006 [50]	Unclear on sample selection and recruitment.	Tools were piloted, male co-researcher engaged to run focus groups and interviews with boys. Does not seem to have been follow-up of findings but did triangulate using FGDs/individual interviews.	Analytical method described, some diversity of perspective explored, particularly through separate gender focus groups.	Findings supported by pseudonymised quotes, including age.	Several topics explored deeply.	Spoke to both boys and girls, but as the focus was on menstrual discourse (not experience) this was important. Unclear whether students/parents had to provide consent/assent to participate. Did try to put participants at ease, especially with gender-matching FGDs and interviews with facilitators.
	Trustworthiness: Medium				Relevance: Medium<br>More about menstruation being discussed than actual experiences	
Fitzgerald 2015 [51]	Researcher advertised via volunteer websites, looking for women who experience menstrual cycle symptom difficulties. The sample was appropriate for attracting women who experienced menstrual difficulties and wanted to actively share their experiences. The use of hormonal contraceptives was often discussed.	The qualitative interview instrument was piloted with six individuals. There was no follow-up, but the responses to the qualitative interview informed the development of quantitative tools. The combination of the qualitative and quantitative data allowed for triangulation. Informed consent processes were followed, and the researcher made efforts to put individuals at ease.	Qualitative data analysis was systematic and inductive, quantitative statistical analysis was systematic. The researcher discusses diverse cases within the population.	Sufficient quotes used to support findings. The diagrams developed from the qualitative data need more explanation.	In depth analysis of the experiences of individuals across multiple domains impacted by menstrual difficulties.	The researcher was very aware of the potential for biasing her results due to her position as a counsellor and worked to reduce this as much as possible. Women with menstrual difficulties were not directly involved in developing the qualitative research, but the initial qualitative tool was piloted with 6 women experiencing menstrual difficulties. For the quantitative component, women with menstrual difficulties were not directly involved in developing the tool, but it was developed from the responses of women in the qualitative phase.
	Trustworthiness: Medium<br>Sampling not ideal and not a lot of references to literature, but thorough				Relevance: High	
Frank 2020 [52]	Convenience and snowball sampling due to vulnerability of participants. Details on gender, sexuality, age and ethnicity provided. Author noted prevalence of white individuals, likely linked to the location of the study (mostly recruiting from a Midwest university).	Targeted interview questions based on online review, but data collection instrument not provided. Unclear whether it was piloted. Details not given on consent/ethics procedures.	Analysis methods unclear but linked to online review. However, various perspectives detailed in findings with pseudonymised quotes.	Detailed, pseudonymised quotes provided to support findings.	Good depth and breadth across topics and individuals.	Positionality was considered and the data collection tool was specifically developed based on research collecting the voices of trans and non-binary individuals who menstruate. No details on consent/ethics procedures provided.
	Trustworthiness: Medium				Relevance: Medium	
Freidenfelds 2009 [53]	Three "sets" of participants, with snowball sampling. Throughout the book we become aware of the backgrounds of various interviewees. At least some of the time it is mentioned whether the interviewee is on hormonal contraception.	A standard interview guide was used, but each interview was reflexive within itself. There does not seem to have been follow-up.	Unclear how analysis was undertaken	A lot of data provided to support findings, and author claims to only generalise for findings where she found saturation in the data.	Wide range of views on variety of topics.	The author took steps to put participants at ease. Interviews were reflexive. Researcher allowed interviews with women to continue until they felt they had articulated their story appropriately.
	Trustworthiness: Medium				Relevance: High	

Reference ID	1. Rigor in sampling	2.Rigor in data collection	3.Rigor in analysis	4.Findings supported by the data	5.Breadth and depth of findings	6.Privileges perspectives of those who menstruate
George & Murcott 1992 [54]	Sample seemed appropriate, but not random.	Full details in another publication.	Full details in another publication.	Pseudonymised quotes.	Limited.	To some extent.
	Trustworthiness: Low				Relevance: Medium	
Golub & Catalano [55]	Most participants college educated. Recruitment strategy unclear.	No details of open-ended questions provided, no details of participants being able to give consent, etc.	No details of qualitative analysis method provided. No names ascribed to quotes.	Quotes given do support the statistical data and the author's conclusions.	Narrow topic, unclear whether quotes were from a variety of individuals.	Can't tell as method does not detail method development, confidentiality or analysis.
	Trustworthiness: Low				Relevance: Low	
Goolden 2018 [56]	Convenience/purposive sampling of menstrual health professionals in UK and Uganda, to gain insights across larger populations. For this review, can only use data collected from UK interviewees about their own menstrual experiences - although these could be biased by their work in menstrual health and desire to communicate a certain narrative to the researcher.	Unclear whether tools were piloted, but participants did provide informed consent to participate. Research did not follow up but did prompt throughout interviews.	Good overview of analytical technique and exploration of responses.	In relation only to personal experiences of UK participants, which were limited.	In relation only to personal experiences of UK participants, which were limited.	Semi-structured interviews with prompts, informed consent, inductive analysis
	Trustworthiness: Medium				Relevance: Low	
Grundstrom et al. 2018 [57]	Sample small and purposive, but all had laparoscopy-confirmed diagnosis of menstruation, so seems a relevant recruitment strategy and sample size for in-depth qualitative research. No information on specific treatments/hormonal contraceptive usage for individuals.	Interview tool was piloted, but is not available, and it is unclear what questions were asked. Followed ethical procedures for informed consent and interviews were conducted in participants' homes. No follow-up.	Data analysis was systematic and performed by three individuals, diversity was explored between women.	Participants' quotes are numbered, but are limited, and the quotes used often don't directly support the point being made by the authors.	Fair insight into individual responses and comparison across experiences.	Unclear what questions were asked in the interviews. However, analysis was very much inductive, and care was taken to put participants at ease.
	Trustworthiness: Medium				Relevance: Medium	
Hawkey et al. 2017 [58]	Large sample of potentially difficult to recruit population. Reasonable explanation for excluding participants from South-East Asia and China. Interviews continued to saturation for each cultural group.	Clear topic guide for interviews and FGDs piloted, interviews/FGDs were reflexive, ethical procedures in place to ensure informed consent and that participants were as comfortable as possible, particularly using community interviewers and provision of childcare.	Very thorough analytical method detailed and followed.	Good use of supporting quotes.	Range of topics and participants of various backgrounds, with investigation into individual experiences where warranted.	People who menstruate, and those sharing cultural beliefs of participants, involved in piloting and adjusting data collection and interpretation.
	Trustworthiness: High				Relevance: Medium<br>Sub-population of refugees/immigrants	

Reference ID	1. Rigor in sampling	2.Rigor in data collection	3.Rigor in analysis	4.Findings supported by the data	5.Breadth and depth of findings	6.Privileges perspectives of those who menstruate
Jackson 2019 [59]	Small sample, which would have been influenced by self-selection, however, the sample was multi-cultural and important demographic and family details on each participant are given.	Assent and consent procedures were adhered to for all participants. Topics of the interview are given, but it's unclear how reflexive the process was. Does not appear to have been piloted and was no follow-up.	Data analysis was systematic, and authors noted any differences between data and the literature/their preconceptions.	Pseudonymised quotes and literature citations support the findings.	Wide variety of topics around menarche/menstruation discussed and presented for each woman, although sample size was small.	Unclear how reflexive the interview schedule was, and it does not seem to have been developed/piloted with the target population. Analysis was inductive though, and steps were taken to sure confidentiality.
	Trustworthiness: Medium				Relevance: Medium	
Jackson & Falmagne [60]	A small sample, but authors do note that its homogeneity is representative of the college where they were recruited from.	Data tools were piloted, ethics committee granted permission (although no details on how informed consent was obtained), interview tool was reflexive.	Data analysis was systematic, and authors noted any differences between data and the literature/their preconceptions.	Pseudonymised quotes support the findings	Wide variety of topics around menarche/menstruation discussed and presented for each woman, although sample size was small.	Young women who menstruate were involved in developing/piloting the research tool, data analysis was inductive. No details of positionality or physical environment/consent procedures
	Trustworthiness: Medium<br>Good study, but very small sample, and a few key bits of info (e.g. positionality and consent procedures) missing.				Relevance: Medium<br>Insightful study, but small sample size and most focus is on menarche.	
Kalman 2003 [61]	Recruitment method is unclear. Girls could be pre- or post-menarcheal and needed to be living with father but not mother. Also, difficult to discern the living situation of each girl. Key demographics in a table would have been useful. Purposive sampling was undertaken to reach saturation. Interviewing a single girl who lived with both parents does not offer a useful comparison.	Informed consent/assent procedures were followed. Researchers did not appear to follow-up. Method does not appear to have been piloted.	Informed consent/assent procedures were followed. Researchers did not appear to follow-up. Method does not appear to have been piloted.	The data presented do illustrate the findings, but there is minimal discussion of the findings considering previous literature.	Fair breadth given the topic of investigation	Unclear how the research was developed. Analysis was inductive. Researchers do not discuss their positionality. Informed consent/assent procedures were followed.
	Trustworthiness: Medium<br>The findings backed up by quotes are trustworthy, just limited interpretation				Relevance: Medium<br>Low conceptual depth and sub-population- but supports menstrual experience narratives more generally	
Kalman 2003 [62]	Recruitment method is unclear. Girls could be pre- or post-menarcheal and needed to be living with father but not mother. Also, difficult to discern the living situation of each girl. Key demographics in a table would have been useful. Purposive sampling was undertaken to reach saturation.	Informed consent/assent procedures were followed. Researchers did not appear to follow-up. Method does not appear to have been piloted.	Analysis followed grounded theory methods, but it is unclear whether this was systematic. Contrasting perspectives of girls were considered. Limited references to previous literature to interpret findings.	The data presented do illustrate the findings, but there is minimal discussion of the findings considering previous literature.	Fair breadth given the topic of investigation.	Unclear how the research was developed. Analysis was inductive. Researchers do not discuss their positionality. Informed consent/assent procedures were followed.
	Trustworthiness: Medium<br>The findings backed up by quotes are trustworthy, just limited interpretation				Relevance: Medium<br>Low conceptual depth and sub-population- but supports menstrual experience narratives more generally	

Reference ID	1. Rigor in sampling	2.Rigor in data collection	3.Rigor in analysis	4.Findings supported by the data	5.Breadth and depth of findings	6.Privileges perspectives of those who menstruate
Kissling 1996 [63]	Unclear how the sample was defined before recruitment, but details on the girls and mothers included is given.	Used flexible interview protocols that have been successful in previous studies, spoke to interviewees alone/with mothers/with other girls dependent upon their preference. Participants asked later whether they concurred with findings.	Detailed analytical procedure described.	Data is presented using pseudonyms, and quotes support the findings.	Depth in exploring individual experiences, but topic focussed on menarche, particularly preparation for it.	Gave participants options for who would be present during the interview. Participants weren't involved in designing the data collection or analytical methods, but their feedback was sought on the authors' findings.
	Trustworthiness: High				Relevance: Medium	
Koutroulis 2001 [64]	Group of 8 women, largely known to author.	Data collection tools weren't piloted, although do seem to have been reflexive, and the method leant itself to follow-up/co-analysis with participants.	Unclear whether analysis was systematic. Diversity in perspective was explored, but it's unclear whether some of the findings have been guided by pre-conceptions or over-analysing the data.	Some quotes presented, but it's unclear whether some of the findings have been guided by pre-conceptions or over-analysing the data.	Very small group and majority of the discussion presented is about menstruation as "dirty" or "clean".	Women who menstruate were involved in providing and. analysing the data
	Trustworthiness: Low				Relevance: Low	
Lee 1994 [65]	Volunteers recruited via word of mouth, but representative of cultural make-up of the university. Few details given on individuals (beyond country of origin).	Data tools not provided and very limited description of the interview/written narrative process. No details of consent processes.	Can't tell whether the analysis was systematic, but diverse opinions were explored.	Some quotes are identified by name/cultural identity, but not all, so difficult to discern cultural patterns.	Limited topic coverage as menarche, but depths into individual studies.	Analytical method used (phenomenological) suggests induction in the analysis. Unclear on other aspects of privileging voices.
	Trustworthiness: Low				Relevance: Medium<br>Wide range of women, but menarche focussed	
Lee 2008 [66]	Large sample size for a qualitative study - self-selecting, but necessary for this scale of study. Participants were all enrolled in a gender course, so may have divergent views from wider population.	Data collection was methodical and allowed participants to provide a vivid description. However, does not seem to have been piloted and consent procedures not reported. Students were assured that participation was not linked to their grades.	Analysis was rigorous and alternative viewpoints and explanations were sought out.	Sufficient identified quotes supported findings.	Good breadth and depth, although the topic was limited.	Narrative method allowed participants to take part in the comfort of their own homes, and open-ended questions allowed them to express their responses as desired. Coding was inductive.
	Trustworthiness: High				Relevance: Medium<br>Menarche specific	
Lee 2009 [67]	Large sample size for a qualitative study - self-selecting, but necessary for this scale of study. Participants were all enrolled in a gender course, so may have divergent views from wider population.	Data collection was methodical and allowed participants to provide a vivid description. However, does not seem to have been piloted and consent procedures not reported. Students were assured that participation was not linked to their grades.	Analysis was rigorous and alternative viewpoints and explanations were sought out.	Sufficient identified quotes supported findings.	Good breadth and depth, although the topic was limited.	Narrative method allowed participants to take part in the comfort of their own homes, and open-ended questions allowed them to express their responses as desired. Coding was inductive.
	Trustworthiness: High				Relevance: Medium<br>Menarche specific	

Reference ID	1. Rigor in sampling	2.Rigor in data collection	3.Rigor in analysis	4.Findings supported by the data	5.Breadth and depth of findings	6.Privileges perspectives of those who menstruate
Lee & Sasser-Coen 1996[68]	Attempt was made to recruit sample via snowball sampling and nursing homes. Suitable for this study's objective (explore, don't generalise). Most women were white, but this is representative of Oregon population. Several demographic details of women shared.	Details of the content of data collection tools described and allowed participants to be reflexive. No follow-up. Several steps taken to ensure participants were comfortable.	Can't tell whether the analysis was systematic, but diverse opinions were explored.	Quotes are identified to specific women/ages, sufficient data illustrates the findings.	Limited topic coverage as menarche, but depths into individual studies.	Analytical method used (phenomenological) suggests induction in the analysis. Unclear on other aspects of privileging voices.
	Trustworthiness: Medium				Relevance: Medium	
Lee 2002 [69]	Participants all volunteered to be interviewed with regards menstruation, but recruitment method is unclear. Some demographic details are provided. Nothing on the use of hormonal contraception.	Informed consent procedures were followed. Researchers followed-up with participants to validate their findings. Data collection tools do not appear to have been piloted.	Data analysis was systematic and explored opinions of women with both positive and negative views of menstruation. Researchers followed up to validate findings.	Several quotes support each finding presented by authors. Quotes had pseudonyms attached.	Variation between participants noted, reasoning behind participants' beliefs, etc., are explored.	Results were inductive and informed consent procedures followed. Unclear whether those who menstruate designed the research.
	Trustworthiness: Medium				Relevance: Medium<br>Focus is mostly on PMS	
Li et al. 2020 [70]	Participants within targeted age group purposively recruited from existing patients until saturation was reached.	Unclear whether the instrument was pre-tested. List of topics provided but not actual data collection tool. Attempts were made to put participants at ease, particularly those whose parents/guardians had accompanied their interview.	Coded using constant comparative technique, illustrative quotes for codes provided.	Numbered quotes provided to support each finding.	Views of a variety of participants represented with some insight to depth.	Unclear, although analysis was inductive.
	Trustworthiness: High				Relevance: Medium	
Lowik 2020 [71]	Broad purposive and snowball sampling, appropriate to reaching this population	Unclear whether data tools were piloted, but multiple, reflexive methods were used, and effort was made to build rapport and comfort with participants.	Analysis was systematic and explored diversity in perspectives.	Findings are grounded in data, and author is clear that the findings are indicative of individuals, not generalisable.	Depth of issues explored for individual participants, difficult to compare across sample.	Positionality of researcher (who menstruates) considered in interpretations. Efforts to put participants at ease and allow for open ended responses
	Trustworthiness: High				Relevance: Medium	
Marshall 1998 [72]	Women experiencing menstrual loss without other pathology referred by GP. Unclear what treatment women had had in the past or whether they were taking hormonal contraceptives.	Steps were taken to ensure participants were comfortable and informed consent process was followed. The interview was reflexive. It is unclear whether it was piloted.	Data analysis was systematic, diversity in opinions explored.	Quotes to support most findings, although they are not numbered.	A range of physical and psychological issues covered. Difficult to discern how many individuals are quoted as they are not numbered.	The researcher was very reflexive in her research topic and within interviews and considers her own positionality throughout.
	Trustworthiness: Medium				Relevance: Medium	
Marshall et al 2019 [73]	Sample was appropriate to the study aims.	Unclear.	Very limited details of qualitative analysis method.	Quotes not provided beyond individual ‘keywords’.	Limited breadth or depth.	Unclear.
	Trustworthiness: Low				Relevance: Low	

Reference ID	1. Rigor in sampling	2.Rigor in data collection	3.Rigor in analysis	4.Findings supported by the data	5.Breadth and depth of findings	6.Privileges perspectives of those who menstruate
Mason & Cunningham 2008 [74]	Recruited mothers and daughters via Down syndrome groups/associations. 6 women with Down syndrome interviewed, details not given on their age, location, etc.	Informed consent processes were followed, data collection tool was developed by a group of women with Down Syndrome, or with family members with Down Syndrome. Data collection was reflexive, with the interview process being improved over time. There was no follow-up.	Data analysis was systematic and explored a diversity of narratives.	Several quotes given and identified as either "mother" or "daughter". Beyond that, demographics of interviewees providing each quote is unclear	Investigated a variety of menstrual factors but lacked depth.	Data collection tool was developed by a group of women with Down Syndrome, or with family members with Down Syndrome. However, most interviews were with mothers of women with Down Syndrome
	Trustworthiness: Medium				Relevance: Low	
Matías-González et al. 2020 [75]	Women with endometriosis were recruited from multiple municipalities to avoid biasing women living in the capital. Use of hormonal contraceptives not discussed. Limited demographics beyond location of focus groups provided.	Interview tool provided, unclear whether it was piloted. Only one focus group conducted, and participants not contacted afterwards. Ethical approval was granted, but few details on where FGDs were or how participants were made to feel comfortable.	Data analysis was systematic and consensus on all coding and themes was required (i.e. 100% inter-rater reliability). Not a lot of diversity in opinion provided, although there may have been limited diversity in opinion!	Quotes provided support the findings but are only differentiated by the location of the FGD (not individuals).	Fair depth into individual stories but focused mostly on one recurring experience.	Unclear how the interview guide was developed and who was involved. Positionality of researchers not discussed. Confidentiality ensured by not collecting personal details. Analysis was inductive.
	Trustworthiness: Medium				Relevance: Medium	
McKechnie 2000 [76]	The abstract tells us that the interviews were conducted with 29 women who had consulted a GP about menstrual irregularities, but there is nothing in the main text about how this recruitment took place. Some of these women had grown up in LMICs, but it is generally impossible to discern which from quotes.	Very little on the data collection methodology	No details of analytical method given	Very few quotes to support the author's findings.	Some depth to individual stories, but the lack of data presented means that breadth cannot be discerned.	Women who menstruate are the interviewees.
	Trustworthiness: Low				Relevance: Low	
Moas 2010 [77]	Self-selecting group of self-identified "menstruation positive" individuals. But could be justified as the study was looking for experiences of those who "value" their menstruation. None used oral contraceptives (this was not part of study design).	Guides for data collection included as appendices, interviews were reflexive to allow for unexpected themes, participants were informed of all details of the study and provided consent. Followed up with some participants.	Details of analytical method given. Unclear whether findings were triangulated, but some interviewees were followed-up.	Very clear on the characteristics of each participant and who gave each quotation. Ample quotes support the findings.	Depth in exploring each woman's experience. Topic was limited to "positive" menstrual attitudes, but that was the study objective, so would also be comfortable with good for both.	Unclear whether women were involved in developing the methodology, but many steps were taken to put participants at ease and consider the positionality of the researcher.
	Trustworthiness: High				Relevance: Medium<br>Specific sample of "menstrual positive" women but does allow us to compare to the dominant discourse of periods as negative experiences.	
Murray 1997 [78]	No mention of recruitment.	Data collection instrument or overview of interview questions not provided.	No analytical method mentioned.	Quotes provided in most cases to support findings.	Comparison of individual views/experiences.	Mostly unclear, but quotes are given from menstruating women to illustrate findings.
	Trustworthiness: Low				Relevance: Medium	

Reference ID	1. Rigor in sampling	2.Rigor in data collection	3.Rigor in analysis	4.Findings supported by the data	5.Breadth and depth of findings	6.Privileges perspectives of those who menstruate
Murray 1998 [79]	No mention of recruitment.	Data collection instrument or overview of interview questions not provided.	No analytical method mentioned.	Quotes provided in most cases to support findings.	Comparison of individual views/experiences.	Mostly unclear, but quotes are given from menstruating women to illustrate findings.
	Trustworthiness: Low				Relevance: Medium	
Murray 1996 [80]	Snowball and purposive sampling through personal and professional networks to obtain a range of ages and ethnicities. Sampling strategy due to topic being so sensitive it was difficult to recruit participants willing to talk about it. Details of each interviewee, including age, ethnic background, etc, are given.	Interview tool provided is comprehensive to the research questions. A short discussion of ethically presenting stories is included, but no details of formal consent processes or how interviews were conducted (e.g. time, place). It is unclear whether the tool was piloted, although it was refined throughout the course of the research.	The analysis itself is very thorough, triangulating across multiple sources, but the analytical method is not entirely clear.	Findings supported by quotes attributed to individual interviewees.	Several topics explored deeply.	The researcher asked questions that were open ended and allowed for interviewees to explore their own answers in real time. She considered positionality throughout, particularly around her interpretation of interviewees' stories. The analytical framework is unclear, so it is difficult to discern between a priori and inductive analysis.
	Trustworthiness: Medium				Relevance: High	
Newton 2012 [81]	Recruitment is unclear.	Unclear, although ethical approval was given by University.	Unclear	Quotes given including numbered pseudonyms.	Focus is mostly on discourse.	Privileges voices of menstruators.
	Trustworthiness: Low				Relevance: Medium<br>Focuses mainly on discourse and less on experiences during menses	
Newton 2016 [82]	Not designed to be representative but did try to get as many people to fill out questionnaire as possible.	Interview could be considered "following up" from questionnaire (author knew their answers). Can't tell whether piloted, but as semi-structured I assume she adapted as she went.	Diversity in perspective was explored, but it's unclear whether the methods were systematic -this is probably clear in her thesis but not here as this is a book for audiences who might not be so interested. Need to see if it is discussed more in the paper we're including (if they are the same population) - checked paper and it has even less on analysis methods!	Many quotes given including numbered pseudonyms.	Focus is mostly on discourse.	Privileges voices of menstruators.
	Trustworthiness: Medium				Relevance: Medium<br>Focuses mainly on discourse and less on experiences during menses	
O'Flynn &Britten 2000 [83]	Participants were recruited via GPs after consulting about heavy periods. It's unclear which participants were taking hormonal contraceptives.	Data collection instrument was developed in consultation with women who experience heavy bleeding, and piloted. Researchers followed up with interested participants to confirm their findings. Ethical consent processes were followed.	Diversity of perspective was explored. Unclear how systematic analysis was as it is described as "The `framework' method of content analysis was used to categorise themes". Interviews continued until saturation was reached.	Quotes support the findings and represent diverse experiences. Quotes are numbered to identify participants.	View on multiple aspects of heavy bleeding and fair depth in individual experiences	Data collection instrument was developed in consultation with women who experience heavy bleeding, and piloted. Researchers followed up with interested participants to confirm their findings. Ethical consent processes were followed.
	Trustworthiness: High				Relevance: High	

Reference ID	1. Rigor in sampling	2.Rigor in data collection	3.Rigor in analysis	4.Findings supported by the data	5.Breadth and depth of findings	6.Privileges perspectives of those who menstruate
O'Flynn 2006 [84]	The sample was recruited via various services. The sample was representative of the ethnic make-up of central London. Recruitment for both sets of interviews continued until important themes were saturated. Sample was likely biased towards women who regularly attended various primary services/women's centres (so may have had less menstrual/health complaints generally).	The data collection instrument was piloted during the first round of surveys. The majority of interviews took place in interviewees' homes. Ethics permission was granted by local Boroughs.	The only details of the analytical method given are that it was "constant comparative analysis", but authors do explore diversity in perspective.	Findings supported by quotes, including pseudonym, age, and menstrual complaint.	Multiple views considered, fairly representative sample for a small qual study.	Coding was inductive. Informed consent procedures were followed. Positionality not mentioned, but menstruating women were involved in piloting the work.
	Trustworthiness: High				Relevance: High	
Oinas 1999 [85]	Participants were appropriate given the author's caveats that the study is specifically about highly educated, middle-class, Finnish women. There is no information given on how they were recruited.	Participants were data providers, collectors and analysts. By the nature of the study they would have been able and willing to participate. However, it is unclear whether the tools (for the narrative and FGD) were piloted, and if they had been, how they would have been evaluated.	It is unclear whether the data analysis methods were systematic; the author does not elaborate on what happened during the group analysis sessions, or how the individual analysis was conducted.	Very few quotes given to support findings, and where they are given, they are often insufficient to demonstrate the findings and back-up the literature being cited.	There seems to have been the potential for a lot of depth, but it isn't presented in the publication.	Middle-class Finns who menstruate were data providers, collectors and analysts.
	Trustworthiness: Low				Relevance: Low	
Owen 2020 [86] (Australia)	Sampling was purposive and convenience-based and included snowball sampling. The participants were current students or recent graduates of a University, who self-selected to be involved, so is biased to quite liberal, young women (but the author does not hide from this fact). Unclear which participants were taking hormonal contraceptives.	Data collection was comprehensive and flexible, including both individual interviews and diaries, with the collection of regular fieldnotes by the author. The data collection instruments were modified as appropriate as data collection continued.	Data analysis was systematic and considered a diversity in perspective.	Pseudonymised quotes provided as evidence for all findings.	Good breadth and depth – deep investigations at participant level and a diversity of experiences explored across the sample.	The author's positionality was frequently assessed and considered regarding the data collection and analysis. Participants were involved in designing the data collection tools once they had been recruited.
	Trustworthiness: High				Relevance: High	
Owen 2020 [86] (UK)	Word of mouth of employees of a small company developing/implementing a menstrual leave policy. Use of hormonal contraceptives unclear.	Data collection was comprehensive and flexible, including both individual interviews and team meetings, with the collection of regular fieldnotes by the author.	Data analysis was systematic and considered a diversity in perspective.	Pseudonymised quotes provided as evidence for all findings.	Focus on menstruation in the workplace, but fair comparison between participants and their differing experiences.	The author's positionality was frequently assessed and considered regarding the data collection and analysis.
	Trustworthiness: High				Relevance: Medium<br>Mostly focused on the policy design/implementation rather than individual experiences	

Reference ID	1. Rigor in sampling	2.Rigor in data collection	3.Rigor in analysis	4.Findings supported by the data	5.Breadth and depth of findings	6.Privileges perspectives of those who menstruate
Pafford 2007 [87]	Small sample for interviews, but purposively selected to include "extremes" (particularly after issues with convenience sampling during pilot). Tried to recruit more men but were generally unwilling to engage/consent (many did engage in informal conversations during participant observation).	Data collection tool was piloted, and several improvements made. Researcher did not interview anyone within her line of command, and no interviews took place with either person in uniform. Informed consent and ethical guidance followed. Two interviews were included from the pilot, slightly concerning as the interview protocol did change significantly.	Data analysis methods were systematic. Little diversity in perspective was explored, except for the single male participant. It is unclear whether the findings were guided by preconceptions or the data, and alternative explanations were not investigated.	Some quotes provided, but they aren't numbered.	A small sample size, and as quotes aren't numbered, it's unclear how generalisable the findings are. There is very little on menstruation despite it being one of the core topics of interest, the main focus was on the time taken to toilet during convoys and how to keep one's gear clean whilst using a porta-loo.	Difficult to tell.
	Trustworthiness: Medium- design seems very good, but reporting not done well				Relevance: Low	
Pascoe 2007 [88]	Sample is small (12 women) and all are known to the author - which she states is because she wants them to feel comfortable discussing such a sensitive topic. Several details given on each interviewee, including for some whether they are taking hormonal contraceptives.	Data collection method not detailed beyond "oral histories", although author did particularly interview women, she knew to put participants "at ease".	No real "analysis", really just reporting on themes of silence from interviews.	Good grounding because not really any interpretation - so quotes and findings are the same.	Only a small amount of information presented for each woman, all on silences around menstruation.	Only women who menstruate were interviewed, and the interviews are said to be reflexive, but unclear how the method was developed and whether there was any analysis (paper is predominantly a summary of responses).
	Trustworthiness: Medium<br>Not a large or deep study, but the quotes from women who menstruate and their experiences with "silence" are shared.				Relevance: Low<br>Some relevance, but only a small amount of data presented, and not interpreted to larger findings (other than that menstruation has been talked about more since second wave feminism)	
Pascoe 2015[89]	Sample is small (13 women - must have interviewed another woman since Pascoe 2007 paper) and all are known to the author - which she states is because she wants them to feel comfortable discussing such a sensitive topic. Several details given on each interviewee, including for some whether they are taking hormonal contraceptives.	Data collection method not detailed beyond "oral histories", although author did particularly interview women, she knew to put participants "at ease".	No details given of analytical method. Quotes from oral histories compared to menstrual products and advertising of the time to demonstrate how experiences were influenced by social norms.	Quotes and details of products/advertising generally in line, although at some points there may be some author interpretation without much literature to back up the links.	Delves into experiences of each woman and compares to contemporary products/advertising, but a very small sample over a large time period.	Only women who menstruate were interviewed, and the interviews are said to be reflexive, but unclear how the method was developed and whether there was any analysis.
	Trustworthiness: Medium<br>No details of analytical methods, but does present data to back up findings in most instances				Relevance: Medium<br>A small study, but with in-depth details of experiences of the participants.	
Patterson & Hale 1985 [90]	Snowball and purposive interviewing as grounded theory emerged. No information on individual participants.	Unclear how the methodology was developed but appears to have been through consideration of literature on developing grounded theory.	Analysis was systematic, but details not given of codes identified, etc.	Limited use of evidence to support findings.	Breadth of findings, depth linked to existing literature.	Only women who menstruate interviewed, but unclear otherwise.
	Trustworthiness: Medium				Relevance: Medium	

Reference ID	1. Rigor in sampling	2.Rigor in data collection	3.Rigor in analysis	4.Findings supported by the data	5.Breadth and depth of findings	6.Privileges perspectives of those who menstruate
Prileszky 2013 [91]	Purposeful selection of women enrolled in an RCT for women experiencing heavy menstrual bleeding.	Data collection method piloted, informed consent from all participants, locations and times of interviews scheduled to put interviewees at ease and reduce disruption on their lives.	Data analysis process clearly detailed; findings reviewed with participants.	Names assigned to quotes, sufficient quotes to support findings.	Investigation of several experiential factors with a range of women (due to purposeful recruitment).	Women weren't involved in developing the study, but the study was piloted to ensure it would gather relevant information on their own experiences and several steps were taken to ensure they were comfortable voicing their own opinions and discussing sensitive topics during interviews.
	Trustworthiness: High				Relevance: Medium<br>Specific condition, but lots on general experiences as well	
Raynor 2020 [92]	Very small sample, although logistically appropriate to a Bachelors research project (2 in-depth interviews each with 3 participants). Attempted to recruit further, but attributes non-recruitment to stigmatised topic. Demographic details provided for three interviewees.	Limited details of data collection tool beyond list of topics discussed. Unclear whether it was piloted. Ethical approval was given for the study, and interviewer specifically took verbal consent to avoid documenting the names of participants.	Analysis was systematic.	Although the sample size is small, the findings are backed up by 2-3 (pseudonymised) participants for each finding.	The opinions and experiences of the three participants are investigated.	Data collection tool not provided, but clearly interviews were reflexive, and the researcher considered her positionality throughout.
	Trustworthiness: Medium				Relevance: High	
Rodgers 2001 [93]	21 is a good sample size, not sure there was a need to have geographic range with such a small number though; actually, dilutes the research findings.	Use of trusted intermediaries, interviews in participants' homes, tool developed with advisory committee and two women with intellectual disabilities, tool piloted.	Some description of analytical method, and attempt to privilege voices of participants during analysis, including visiting multiple times and allowing for feedback to the findings.	Findings clearly supported by the data presented and names are attributed to individual quotes.	Presents in-depth discussions with women across many facets of menstrual experience (i.e. not just using pads).	Women with intellectual disabilities involved in designing the research, and researcher went to great lengths to hear from women themselves (rather than carers)
	Trustworthiness: High				Relevance: Medium<br>Insightful for menstruators with intellectual disabilities	
Rubinsky et al. 2020 [94]	Purposive sampling via authors' social media accounts. Characteristics of sample provided.	Unclear whether the tool was piloted, but it was flexible enough for participants to complete in their own time and with open-ended responses. IRB ethical approval was granted. There was no follow-up.	Data analysis was systematic and involved all authors. A diversity of perspectives was explored.	Many number quotes provided to support findings.	Wide range of perspectives presented with evidence.	The positionality of researchers and who developed the survey are unclear. Responses were open ended and allowed menstruators to share as little or as much as they wanted to. Confidentiality was assured.
	Trustworthiness: High				Relevance: High	
Rydström 2018 [95]	Convenience and snowball sampling due to vulnerability of participants. Details on gender, age and menstrual status provided. Details of hormonal contraceptives of or other hormones clear for each participant.	A semi-structured interview instrument was developed, but author specifically aimed to let participants go 'off topic' to discuss the topics they thought were most pertinent to menstrual experiences whilst non-binary or trans. Only one method of data collection used.	Data analysis was systematic, and codes are provided (although not definitions). Diversity in perspectives were explored.	Pseudonymised quotes provided for all findings.	Good breadth of topics across participants and depth investigating individual experiences.	Participants 'drove' interview topics, steps were taken to put them at ease, the positionality of the researcher was considered throughout design, data collection and analysis.
	Trustworthiness: High				Relevance: High	

Reference ID	1. Rigor in sampling	2.Rigor in data collection	3.Rigor in analysis	4.Findings supported by the data	5.Breadth and depth of findings	6.Privileges perspectives of those who menstruate
Santer 2005 [96]	Random sampling of women followed by purposive sampling of those reporting heavy bleeding, followed by further purposive sampling for individual interviews. Where relevant, use of hormonal contraceptives was mentioned.	Focus groups were held to inform individual interview guides. Topics of interviews detailed and allowed for open-ended discussion. Ethical procedures for informed consent were followed. Interviews and questionnaires were followed up.	Data analysis was clear and systematic, divergent experiences were noted and explore.	Quotes with identifiers support findings, further backed up by literature sources.	Open ended interviews about a range of topics with several women.	Women who menstruate were involved in developing the research and the author reflects on her own positionality during analysis.
	Trustworthiness: High				Relevance: High	
Santer et al. 2007 [97]	Random sampling of women followed by purposive sampling of those reporting heavy bleeding, followed by further purposive sampling for individual interviews. Where relevant, use of hormonal contraceptives was mentioned.	Focus groups were held to inform individual interview guides. Topics of interviews detailed and allowed for open-ended discussion. Ethical procedures for informed consent were followed. Interviews and questionnaires were followed up.	Data analysis was clear and systematic, divergent experiences were noted and explore.	Quotes with identifiers support findings, further backed up by literature sources.	Open ended interviews about a range of topics with several women.	Women who menstruate were involved in developing the research and the author reflects on her own positionality during analysis.
	Trustworthiness: High				Relevance: High	
Santer et al. 2008 [98]	Random sampling of women followed by purposive sampling of those reporting heavy bleeding, followed by further purposive sampling for individual interviews. Where relevant, use of hormonal contraceptives was mentioned.	Focus groups were held to inform individual interview guides. Topics of interviews detailed and allowed for open-ended discussion. Ethical procedures for informed consent were followed. Interviews and questionnaires were followed up.	Data analysis was clear and systematic, divergent experiences were noted and explore.	Quotes with identifiers support findings, further backed up by literature sources.	Open ended interviews about a range of topics with several women	Women who menstruate were involved in developing the research and the author reflects on her own positionality during analysis.
	Trustworthiness: High				Relevance: High	
Sasser-Coen 1997 [99]	Attempt was made to recruit sample via snowball sampling and nursing homes. Suitable for this study's objective (explore, don't generalise). Most women were white, but this is representative of Oregon population. Several demographic details of women shared.	Data collection tools not piloted, and no follow-up. Data collection tools included in appendix, and author went to significant lengths to ensure oral and written histories were similar and that participants were comfortable with the time and place of the interview, including with informed consent procedures.	Data analysis was systematic and explored diverse opinions.	Ample quotes presented and attributed to individuals.	Broad and deep findings.	Older women who menstruate weren't involved in design and there was no follow up. However, author went to great lengths to ensure comfort and informed consent, and discussed her own positionality.
	Trustworthiness: High				Relevance: Medium	

Reference ID	1. Rigor in sampling	2.Rigor in data collection	3.Rigor in analysis	4.Findings supported by the data	5.Breadth and depth of findings	6.Privileges perspectives of those who menstruate
Scott 2020 [100]	Convenience and snowball sampling to recruit women with CPGPCs. Demographic details of interviewees provided.	Unclear whether the interview instrument was piloted. Specific instrument not provided, but details of various topics discussed given. Steps were taken to ensure participants were comfortable with the physical/online setting of the interviews and through disclosing the CPGPC experiences of the interviewers.	Data analysis methods were systematic, but it is unclear whether alternative explanations were sought for findings.	Multiple pseudonymised quotes or examples provided for findings	Limited breadth or depth with regards CPGPCs during menses.	Efforts were made to consider the positionality of researchers and put participants at ease. There was a balance between a priori coding and induction in the analysis.
	Trustworthiness: Medium				Relevance: Low<br>Difficult to discern which results are about pain during menses and pain during other times in the menstrual cycle (only coded experiences which were clearly menses-related)	
Sebert Kuhlmann et al 2019 [101]	Service organizations that were purposively selected to reflect a range of housing shelter (six), food (two), employment and training (one), and drop-in centre (one) services available to low-income women	The survey instrument was pilot tested and revised (although it does not seem to be available). Data collection methodology was determined in coordination with each organization to minimize service disruption to their clients. Informed consent procedures were followed.	The quantitative analysis method was systematic, but there is no description given for qualitative analysis - qualitative findings are more summaries that support the quantitative findings, rather than analytical reflections/generalisations.	Adequate quantitative and qualitative results presented to support findings.	Explored a range of issues and delved into the reasons behind various behaviours, supported by literature	The comfort of participants was front of mind, and interviews, although mostly quantitative, were reflexive to some extent. Women experiencing homelessness were not involved in design of the study and there was no follow-up (admittedly this would have been difficult for this population). However, results were triangulated through three methods of data collection.
	Trustworthiness: High				Relevance: Medium<br>Sub-population and mostly quantitative results	
Secor-Turner et al. 2020 [102]	Convenience sampling to purposively recruit participants within the desired age range. Demographics on age and ethnicity provided.	Unclear whether data instrument was piloted. Procedures for assent and consent were followed.	Data analysis was systematic and by two authors.	Sufficient quotes provided; however, they were not pseudonymised to individuals or which of the three focus groups they came from.	Good depth but further breadth on some topics would have been useful.	The focus group discussions were open-ended, but it is unclear as to how reflexive they were. It is unclear how confidentiality was assured, especially as the girls in each group knew one another. Positionality of the authors is not discussed.
	Trustworthiness: Medium				Relevance: Medium	

Reference ID	1. Rigor in sampling	2.Rigor in data collection	3.Rigor in analysis	4.Findings supported by the data	5.Breadth and depth of findings	6.Privileges perspectives of those who menstruate
Seear 2009 [103]	Snowball sampling through friends, colleagues and advert. Was specifically looking for endometriosis sufferers, so this had some merit.	Six topics for interviews stated, although it is unclear whether these were defined in advance of the first interview. No data collection tool is available. Ethical procedures for informed consent were followed. Researchers did not follow-up a second time or share findings for feedback.	Data analysis procedure given in detail, and involved iterative induction of themes, whilst also taking note of alternative experiences to the bulk of participants.	Quotes (with names and ages) given to support findings, alongside interpretations from past literature.	Quotes allowing for in-depth understandings of the experiences of participants given. Focused mostly on diagnostic delay.	The design of the research study is unclear, but it does seem that participants were given ample opportunity to discuss issues of importance to themselves, and the analysis of the data was iterative (although there was not follow-up). Ethical procedures for informed consent were followed.
	Trustworthiness: Medium				Relevance: Medium	
Segal 1998 [104]	Sample is only 5 individuals, known to author. Did all have diagnosed endometriosis. Author states that she reached saturation with this number, but surely saturation is only within this very small group of individuals. Few other demographics provided.	Author ensured participants were willing to contribute, particularly through an informed consent procedure and the use of written narratives in the first instance. There was some "follow up", but only for clarification of written narratives. Instrument does not appear to have been piloted.	Data analysis methods were systematic. It is unclear whether the analysis was balanced between the data and pre-conceptions as quotes are rarely offered throughout the presentation of findings; when they are, they are not attributed to individuals.	Some use of quotes in section on clusters, although these are not numbered/generally only one quote given for each cluster.	Fair depth into the experience of individuals, but the sample size is so small it is difficult to understand how broad the analysis was.	The researcher has been diagnosed with endometriosis and considers how this positionality influenced her analysis. Coding was inductive.
	Trustworthiness: Low				Relevance: Low	
Skultans 1970 [105]	Quite systematic, it's just that women from that generation weren't keen to talk.	Can't tell.	Not a lot of quotes/proof of what she has written.	Unclear.	Limited.	Hard to tell, comes across as though the author is voicing her own opinions more than participants'.
	Trustworthiness: Low				Relevance: Low	
Skultans 1988 [106]	I know how she selected the 49-51 age group, but unclear how she selected the wider group.	Can't tell.	Can't tell.	Difficult to tell.	Broad findings.	I think so, but difficult to tell.
	Trustworthiness: Low				Relevance: Low	
Sommer et al. 2020 [107]	Participants who presented as women were purposively recruited to represent a large range of ages (ages were estimated). Limited demographics provided/collected from participants.	Unclear whether data collection tool was piloted before use. Processes for consent were followed and researchers attempted to make participants comfortable. Ethical approval had been given.	Data analysis methods were systematic and involved multiple individuals	Very good grounding of results in data provided by multiple participants.	Good/fair breadth and depth.	Unclear as data collection tool not included, but ethical clearance was obtained, and attempts were made to put participants at ease.
	Trustworthiness: High				Relevance: Medium<br>Many findings specific to lower income groups	

Reference ID	1. Rigor in sampling	2.Rigor in data collection	3.Rigor in analysis	4.Findings supported by the data	5.Breadth and depth of findings	6.Privileges perspectives of those who menstruate
Statham 2020 [108]	Majority of the study was quantitative, with elite athletes recruited from a high-performance training environment. For qualitative section, 4 athletes were chosen, with one whose (quantitative) performance was the most affected by menstrual cycle and another whose was the least. Authors are clear on the use of hormonal contraceptives. Demographics of 4 interviewees not provided beyond sporting status.	Data collection topics provided (not specific tool) but was stated to be open-ended. Unclear whether it was piloted. Steps were taken to use an interview technique which built rapport between the interviewer (a peer of interviewees). Ethics procedures followed.	Results of qualitative analysis summarised as a table, so although analysis method is presented as systematic, it is unclear whether this was the case with limited quotes / evidence of probing and preconceptions.	Quotes not provided, just summary of researchers' interpretations.	Limited breadth and depth (very small qualitative sample size and quotes not provided).	Difficult to discern for qualitative component of study.
	Trustworthiness: Medium				Relevance: Low<br>Mostly quantitative study	
Steward et al. 2018 [109]	Sampling strategy was appropriate, although there would have been self-selection for involvement.	Ethics procedures were followed, but it's unclear whether the instrument was piloted.	Little description of analytical method, but appears to have been thematic	Quotes are numbered and support findings.	Quite a large sample, but limited analysis of individual experiences.	A woman with autism was involved in developing the research, alongside other women. Analysis method is unclear.
	Trustworthiness: Medium				Relevance: Low	
Teitelman 2004 [110]	Purposive sampling to achieve representation. Demographic details provided for participants.	Data collection instrument not piloted but reviewed by experts in the fields of adolescent development, family theory, women's health, and qualitative interviewing. Study was approved by an ethics board. Does not appear to have been follow-up. Interviewed until saturation was reached.	Analysis was systematic, and involved first coding "blind" to demographics, followed by using a "Listening Guide".	Data support findings.	Investigated individual narratives of a diverse population through multiple lenses.	Young women who menstruate not obviously involved in designing the research. Data collection instruments not piloted, but reflexive and reviewed by experts in the field. Analysis was inductive. Positionality of research not discussed.
	Trustworthiness: High				Relevance: Medium	
Thuren 1994 [111]	Snowball and purposive sampling. Few characteristics known.	Very little information on data collection method.	Very little information on data collection method, very few quotes so difficult to ascertain bias.	Very little evidence that findings are a result of the primary data.	Very difficult to discern without methodologies or quotes.	Can't tell.
	Trustworthiness: Low				Relevance: Low	
Tingle & Vora 2018 [112]	Say that middle-class white girls are overrepresented. Not statistical though - got mixed schools and made sure to get Catholic and Protestant in Ireland.	Can't tell.	Unclear how data was analysed.	Many quotes provided, some with pseudonyms.	Fair breadth and depths of findings.	Spoke directly to those who menstruate.
	Trustworthiness: Low				Relevance: High	

Reference ID	1. Rigor in sampling	2.Rigor in data collection	3.Rigor in analysis	4.Findings supported by the data	5.Breadth and depth of findings	6.Privileges perspectives of those who menstruate
Tolson et al 2002 [113]	Sampling justified for this study.	Tools were not piloted, but participants were able to participate in many ways, which allowed them to choose the method they were most comfortable with. Ethical procedures for informed consent were followed.	Data analysis described systematically, although it's unclear just how this led to reporting of results. No deviant cases stand out, but authors do note where their findings differ from the literature.	Quotes provided are numbered and support the findings.	Participants' experiences compared and investigated.	Although women with PD who menstruate were not involved in developing the research, a wide range of data collection methods were presented to anyone who wanted to take part, enabling them to contribute to the research in the way they were most comfortable.
	Trustworthiness: High				Relevance: Medium<br>Very specific sub-population, but experiences are similar to wider population	
Trego 2007 [114]	Small sample size, but recruitment ceased when saturation was considered to have been reached by two researchers.	Ethical procedures for informed consent were undertaken. Unclear whether interviews were piloted. Questions were open-ended. There was no follow-up.	Data analysis was systematic and included peer-review by experts.	Thematic synthesis supported by quotes.	For this study, covered a range of issues and responses of multiple women.	Ethical informed consent procedure was in place. Military women not involved in developing data collection tool. No follow-up.
	Trustworthiness: Medium				Relevance: Medium	
Uskul 2004 [115]	The sampling strategy was justified to the question - sampling for international differences at an international conference makes sense. Experiences were mostly cultural, so not distinguishing those taking hormonal contraceptives will not have imposed much bias.	Data tools, ethical procedure for informed consent.	Diversity in perspective was explored as part of the research and is supported by both quotes and literature.	Often unclear whether results refer to all women or specific cultural groups.	Allowed for depth in exploring individual experiences across a broad range of cultures.	Informed consent procedures followed; open-ended discussions encouraged. Positionality of researcher not mentioned.
	Trustworthiness: High				Relevance: Medium	
Vora 2017 [116]	Small and somewhat non-representative sample but recognise that this is a vulnerable hard-to-reach population and that this was a preliminary study.	No details of how data collection tools were developed/piloted, or what questions were asked and whether informed consent was obtained from participants.	No details of analytical method.	Some generalisations made, but quotes provided in several instances.	Very specific study.	No details of how data collection tools were developed/piloted, or what questions were asked and whether informed consent was obtained from participants. But clearly the views of women experiencing menstruation and homelessness were sought.
	Trustworthiness: Low				Relevance: Low	
Vora 2020 [117]	Small and somewhat non-representative sample but recognise that this is a vulnerable hard-to-reach population and that this was a preliminary study.	No details of how data collection tools were developed/piloted, or what questions were asked and whether informed consent was obtained from participants.	No details of analytical method.	Some generalisations made, but quotes provided in several instances.	Very specific study.	No details of how data collection tools were developed/piloted, or what questions were asked and whether informed consent was obtained from participants. But clearly the views of women experiencing menstruation and homelessness were sought.
	Trustworthiness: Low				Relevance: Low	

Reference ID	1. Rigor in sampling	2.Rigor in data collection	3.Rigor in analysis	4.Findings supported by the data	5.Breadth and depth of findings	6.Privileges perspectives of those who menstruate
Whisnant & Zegans 1975 [118]	Selection of post-menarcheal girls was appropriate to research aims, camp counsellors were self-selecting. Easy to discern pre/post-menarcheal responses.	Interview tool does not seem to have been piloted and isn't available. Unclear how consent/assent was obtained.	No analytical method detailed.	The data illustrate the findings, although perhaps not to the level of interpretation presented in the Discussion.	Depth in individual responses, but focus was mostly on menarche.	Unclear how study was designed and analysed. But view of those who menstruate are at the forefront of findings.
	Trustworthiness: Low				Relevance: Medium	
Wigmore-Sykes et al. 2020 [119]	Convenience sampling, although was a pilot study, so somewhat appropriate. Unclear whether there were attempts to recruit a diverse sample. Limited details on demographics of participants.	This is the pilot study of the data collection tools. Unclear contents of data collection tool. Unclear consent/assent procedures, but states that IRB approval was provided.	Limited detail on analysis.	Unclear, not supported by data/quotes.	Limited breadth or depth.	Can't tell.
	Trustworthiness: Low				Relevance: Low	
Wood et al. 2007 [120]	Sample of students on a sexuality course. The title/research question is about "college-aged" - not "college-enrolled", and these students would be more aware than others at their age.	Tools were piloted with 5 students. Unsure whether these were then included in the final 15. After 15 interviews claim to have reached saturation. Only one data collection method - did probe but didn't follow up.	More details on "member checking, verbatim transcription and peer debriefing" would have been useful	Quotes supplied and attributed to individual interviewees by numbers.	Depth in individual responses, limited sample.	Tools were piloted, quotes demonstrate privileging of menstruators' voices.
	Trustworthiness: Medium				Relevance: Medium	
Wootton & Morison 2020 [121]	Purposively recruited girls who had previously approached school nurse requiring menstrual materials. Demographic characteristics provided.	Unclear whether the data collection tool was piloted. Tool not specifically provided, but the range of topics discussed given and the interview was open-ended. Steps were taken to ensure confidentiality and put participants at ease, including engaging a Māori cultural advisor in study design.	Data analysis was systematic, but it is unclear whether analysis was influenced by preconceptions. Most findings were only supported by one example from the participants, so difficult to know whether a diversity of perspectives was investigated, although there were a few examples of differing perspectives around menstruation as gross and/or natural.	Pseudonymised quotes provided to support findings, but normally only a single quote per finding.	Fair range of experiences, but many only considered from perspective of one participant.	Coding was inductive, although may have been led by previous literature. The positionality of the researchers was considered throughout, and efforts were made to make participants comfortable. A person who menstruate designed the research with input from a cultural advisor.
	Trustworthiness: Medium				Relevance: Medium	

References

1. Rees R, Oliver K, Woodman J, Thomas J. Children's views about obesity, body size, shape and weight: a systematic review. London, UK: EPPI-Centre, Social Science Research Unit, Institute of Education, University of London, 2009.
2. Adams-Matthews H. Pomegranate moon: A study concerning adult women's recollection and transformation of menarche and feminine embodiment through ceremony: Pacifica Graduate Institute (USA); 2009.
3. Allen KR, Goldberg AE. Sexual activity during menstruation: a qualitative study. J Sex Res. 2009;46(6):535-45. doi: 10.1080/00224490902878977. PubMed PMID: 19350441.
4. Allyn K, Evans S, Seidman LC, Payne LA. “Tomorrow, I’ll be fine”: Impacts and coping mechanisms in adolescents and young adults with primary dysmenorrhoea. J Adv Nurs. 2020;76(10):2637-47. doi: 10.1111/jan.14460. PubMed PMID: 2443817799.
5. Amann-Gainotti M. Sexual socialization during early adolescence: the menarche. Adolescence. 1986;21(83):703-10. PubMed PMID: 3812077; PubMed Central PMCID: PMC3812077.
6. Andrews S. The experience of menarche: an exploratory study. J Nurse-Midwifery. 1985;30(1):9-14. doi: 10.1016/0091-2182(85)90208-3. PubMed PMID: 3844033.
7. APS Group Scotland. Access to Sanitary Products Aberdeen Pilot: Evaluation Report. Edinburgh, UK: The Scottish Government, 2018.
8. Armeni E. Menstruation goes public: Aspects of women's menstrual experience in Montreal, 1920-1975 [thesis]: McGill University (Canada); 1997.
9. Armour M, Dahlen HG, Smith CA. More than needles: the importance of explanations and self-care advice in treating primary dysmenorrhea with acupuncture. Evid-based Compl Alt. 2016:1-11. doi: 10.1155/2016/3467067. PubMed PMID: 610566995.
10. Armour MJ. The effectiveness of acupuncture in the treatment of primary dysmenorrhea : A mixed methods study [thesis]: University of Western Sydney (Australia); 2015.
11. Artschwager KM. Meanings of menstruation to Mexican American women. In: Komnenich P, McSweeney M, Noack JA, Elder N, editors. The Menstrual Cycle: Research and implications for women's health. 2. New York, USA: Springer; 1981. p. 114-23.
12. Beausang CC, Razor AG. Young Western women's experiences of menarche and menstruation. Health Care Women Int. 2000;21(6):517-28. doi: 10.1080/07399330050130304.
13. Bishop TA. A qualitative study of young adult women's recollections of menarche [thesis]: California School of Professional Psychology - Berkeley/Alameda (USA); 1999.
14. Bobier L. The Sexualization of Menstruation: On Rape, Tampons, and ‘Prostitutes’. In: Bobel C, Winkler IT, Fahs B, Hasson KA, Kissling EA, Roberts T-A, editors. The Palgrave Handbook of Critical Menstruation Studies: Palgrave Macmillan; 2020.
15. Botello-Hermosa A, Casado-Mejia R. Fears and concerns related to menstruation: a qualitative study from the perspective of gender. Texto Contexto Enfer. 2015;24(1):13-21. doi: 10.1590/0104-07072015000260014.
16. Bransen E. Has menstruation been medicalised? Or will it never happen... Sociol Health and Ill. 1992;14(1):98-110. doi: 10.1111/1467-9566.ep11007176. PubMed PMID: 61313038; 92Z1292.
17. Brantelid IEN, H.; Alehagen, S. Menstruation during a lifespan: A qualitative study of women's experiences. Health Care Women Int. 2014;35(6):600-16. doi: 10.1080/07399332.2013.868465. PubMed PMID: 24313552.
18. Briggs A. ‘Period poverty’ in Stoke-on-Trent, UK: new insights into gendered poverty and the lived experiences of austerity. J Poverty Soc Justice. 2020. doi: 10.1332/175982720X16050132762411.
19. Britton CJ. Learning about ''the curse'' - An anthropological perspective on experiences of menstruation. Women Stud Int Forum. 1996;19(6):645-53. doi: 10.1016/s0277-5395(96)00085-4. PubMed PMID: WOS:A1996VY10000006.
20. Brookes B, Tennant M. Making girls modern: Pakeha women and menstruation in New Zealand, 1930-70. Women's Hist Rev. 1998;7(4):565-81. doi: 10.1080/09612029800200183. PubMed PMID: 61624340; 9918589.

21. Brown N, Knight CJ, Forrest LJ. Elite female athletes' experiences and perceptions of the menstrual cycle on training and sport performance. Scand J Med Sci Spor. 2020;n/a(n/a). doi: 10.1111/sms.13818.
22. Bullo S, Hearn JH. Parallel worlds and personified pain: A mixed-methods analysis of pain metaphor use by women with endometriosis. Brit J Health Psych. 2020;n/a(n/a):e12472. doi: 10.1111/bjhp.12472.
23. Burbeck RW, C. The personal experience of dysmenorrhoea: an interpretative phenomenological analysis. J Health Psych. 2014;19(10):1334-44. doi: 10.1177/1359105313490313. PubMed PMID: 604774746.
24. Burrows A, Johnson S. Girls' experiences of menarche and menstruation. J Reprod Infant Psyc. 2005;23(3):235-49. doi: 10.1080/02646830500165846. PubMed PMID: 57054926; 390882.
25. Byles JE, Hanrahan PF, Schofield MJ. 'It would be good to know you're not alone': The health care needs of women with menstrual symptoms. Fam Pract. 1997;14(3):249-54. doi: 10.1093/fampra/14.3.249. PubMed PMID: 27281211.
26. Cattaneo JS. The relationship between menstrual ideology and practice in the lives of women in south-western Ontario [thesis]: Concordia University (Canada); 2000.
27. Chapple A. Menorrhagia: women's perceptions of this condition and its treatment. J Adv Nurs. 1999;29(6):1500-6. doi: 10.1046/j.1365-2648.1999.01038.x.
28. Chen CX, Draucker CB, Carpenter JS. What women say about their dysmenorrhea: A qualitative thematic analysis. BMC Women's Health. 2018;18 (1) (no pagination)(47). doi: 10.1186/s12905-018-0538-8. PubMed PMID: 620922409.
29. Chou Y, Lu ZJ, Wang FTY, Lan C, Lin L. Meanings and experiences of menstruation: perceptions of institutionalized women with an intellectual disability. J Appl Res Intellect. 2008;21(6):575-84. doi: 10.1111/j.1468-3148.2008.00430.x.
30. Chrisler JC, Gorman JA, Manion J, Murgo M, Barney A, Adams-Clark A, et al. Queer periods: attitudes toward and experiences with menstruation in the masculine of centre and transgender community. Cult Health Sex. 2016;18(11):1238-50. doi: 10.1080/13691058.2016.1182645.
31. Christoforou A. Womanhood, reproduction, and pollution: Greek Cypriot women's accounts of menstruation. Women Stud Int Forum. 2018;68:47. doi: 10.1016/j.wsif.2018.02.006. PubMed PMID: 2086368108.
32. Clark M. Experiences of women with endometriosis: an interpretative phenomenological analysis [thesis]: Queen Margaret University (United Kingdom); 2012.
33. Cooper SC, Koch PB. "Nobody Told Me Nothin": communication about menstruation among low-income African-American women. Women Health. 2007;46(1):57-78. doi: 10.1300/J013v46n01_05. PubMed PMID: 211494900.
34. Costos D, Ackerman R, Paradis L. Recollections of menarche: communication between mothers and daughters regarding menstruation. Sex Roles. 2002;46(1-2):49-59. doi: 10.1023/A:1016037618567. PubMed PMID: 60082045; 200218591.
35. Deforest CA. The bloody truth: A psychological and cultural study of menstruation as lived and experienced by women [thesis]: Duquesne University (USA); 2007.
36. DeMaria AL, Delay C, Sundstrom B, Rehberg A, Naoum Z, Ramos-Ortiz J, et al. "my mama told me it would happen": Menarche and menstruation experiences across generations. Women Health. 2019:No Pagination Specified. doi: 10.1080/03630242.2019.1610827. PubMed PMID: 2019-23846-001.
37. DeMaria AL, Meier SJ, Dykstra C. "It's not perfect but it's mine": Genital self-image among women living in Italy. Body Image. 2019;29:140-8. doi: 10.1016/j.bodyim.2019.03.011. PubMed PMID: 136878473.
38. Denny E, Culley L, Papadopoulos I, Apenteng P. From womanhood to endometriosis: findings from focus groups with women from different ethnic groups. Divers Health Care. 2011;8(3):167-80. PubMed PMID: 919970050; 201201544.
39. Dillaway H, Cross K, Lysack C, Schwartz J. Normal and natural, or burdensome and terrible? Women with spinal cord injuries discuss ambivalence about menstruation. Sex Roles. 2013;68(1-2):107-20. doi: 10.1007/s11199-011-0092-4. PubMed PMID: 1448991643; 201345316.
40. Ditchfield H, Burns J. Understanding our bodies, understanding ourselves: the menstrual cycle, mental health and women with learning disabilities. Learn Disabil Rev. 2004;9(4):24-32. doi: 10.1108/13595474200400035.

41. Donmall K. What it means to bleed: an exploration of young women's experiences of menarche and menstruation. Brit J Psychotherapy. 2013;29(2):202-16. doi: 10.1111/bjp.12016. PubMed PMID: 1417549645; 201315380.
42. Elson J. Menarche, menstruation, and gender identity: retrospective accounts from women who have undergone premenopausal hysterectomy. Sex Roles. 2002;46(1-2):37-48. doi: 10.1023/A:1016085501729. PubMed PMID: 60085019; 200219006.
43. Eriksen W. Facing puberty: Exploring the onset, symptoms and experience of menses in females with Autism Spectrum Disorder [thesis]: University of Pennsylvania (USA); 2016.
44. Fahs B. Sex during menstruation: Race, sexual identity, and women's accounts of pleasure and disgust. Fem Psychol. 2011;21(2):155-78. doi: 10.1177/0959353510396674. PubMed PMID: 919667359.
45. Fahs B. Genital panics: constructing the vagina in women's qualitative narratives about pubic hair, menstrual sex, and vaginal self-image. Body Image. 2014;11(3):210-8. doi: 10.1016/j.bodyim.2014.03.002.
46. Fahs B. There will be blood: women's positive and negative experiences with menstruation. Women Reprod Health. 2020;7(1):1-16. doi: 10.1080/23293691.2019.1690309. PubMed PMID: 141877356.
47. Fernandez-Martinez E, Abreu-Sanchez A, Perez-Corrales J, Ruiz-Castillo J, Velarde-Garcia JF, Palacios-Cena D. Living with pain and looking for a safe environment: A qualitative study among nursing students with dysmenorrhea. Int J Env Res Pub He. 2020;17(18):1-15. doi: 10.3390/ijerph17186670. PubMed PMID: 2005064116.
48. Findlay RJ, Macrae EHR, Whyte IY, Easton C, Forrest Nee Whyte LJ. How the menstrual cycle and menstruation affect sporting performance: experiences and perceptions of elite female rugby players. Brit J Sport Med. 2020;54(18):1108-13. doi: 10.1136/bjsports-2019-101486. PubMed PMID: 631660915.
49. Fingerson L. Agency and the body in adolescent menstrual talk. Childhood. 2005;12(1):91-110. doi: 10.1177/0907568205049894. PubMed PMID: 60535694; 200510559.
50. Fingerson L. Girls in Power: Gender, Body, And Menstruation in Adolescence: State University of New York Press; 2006.
51. Fitzgerald K. Women's Health and the Workplace: The Impact of the Menstrual Cycle [thesis]: Universidade Autonoma de Lisboa (Portugal); 2015.
52. Frank SE. Queering menstruation: trans and non-binary identity and body politics. Sociol Inq. 2020;90(2):371-404. doi: 10.1111/soin.12355.
53. Freidenfelds L. The Modern Period: Menstruation in Twentieth-Century America: Johns Hopkins University Press; 2009.
54. George A, Murcott A. Monthly strategies for discretion: shopping for sanitary towels and tampons. Sociol Rev. 1992;40(1):146-62. doi: 10.1111/j.1467-954X.1992.tb02949.x. PubMed PMID: 61308043; 92Y8084.
55. Golub S, Catalano J. Recollections of menarche and women's subsequent experiences with menstruation. Women Health. 1983;8(1):49-61. doi: 10.1300/J013v08n01_06. PubMed PMID: 6868626.
56. Goolden E. Hidden yet shared: an investigation into experiences of the menstrual taboo across higher and lower income contexts [thesis]: University of Leeds (United Kingdom); 2018.
57. Grundström H, Alehagen S, Kjølhede P, Berterö C. The double-edged experience of healthcare encounters among women with endometriosis: A qualitative study. J Clin Nurs. 2018;27(1-2):205-11. doi: 10.1111/jocn.13872.
58. Hawkey AJ, Ussher JM, Perz J, Metusela C. Experiences and constructions of menarche and menstruation among migrant and refugee women. Qual Health Res. 2017;27(10):1473-90. doi: 10.1177/1049732316672639. PubMed PMID: 1928355484.
59. Jackson TE. Policing a menstrual monster: How working class girls discuss their menstrual reactions and experiences. J Youth Stud. 2019;22(2):153-70. doi: 10.1080/13676261.2018.1492100. PubMed PMID: 2018-31895-001.
60. Jackson TE, Falmagne RJ. Women wearing white: discourses of menstruation and the experience of menarche. Fem Psychol. 2013;23(3):379-98. doi: 10.1177/0959353512473812. PubMed PMID: 1504423631; 201408182.
61. Kalman M. Taking a different path: menstrual preparation for adolescent girls living apart from their mothers. Health Care Women Int. 2003;24(10):868-79. doi: 10.1080/07399330390244275. PubMed PMID: 14742126.

62. Kalman MB. Adolescent girls, single-parent fathers, and menarche. Holist Nurs Pract. 2003;17(1):36-40. doi: 10.1097/00004650-200301000-00008. PubMed PMID: 106871159. Language: English. Entry Date: 20031003. Revision Date: 20150818. Publication Type: Journal Article.
63. Kissling EA. Bleeding out loud: communication about menstruation. Fem Psychol. 1996;6(4):481-504. doi: 10.1177/0959353596064002. PubMed PMID: WOS:A1996VM60500002.
64. Koutroulis G. Soiled identity: memory-work narratives of menstruation. Health. 2001;5(2):187-205. doi: 10.1177/136345930100500203.
65. Lee J. Menarche and the (hetero) sexualization of the female body. Gender Soc. 1994;8(3):343-62. doi: 10.1177/089124394008003004.
66. Lee J. 'A Kotex and a smile': mothers and daughters at menarche. J Fam Issues. 2008;29(10):1325-47. doi: 10.1177/0192513X08316117. PubMed PMID: 61680978; 200832224.
67. Lee J. Bodies at menarche: stories of shame, concealment, and sexual maturation. Sex Roles. 2009;60(9-10):615-27. doi: 10.1007/s11199-008-9569-1. PubMed PMID: 61767324; 200928778.
68. Lee J, Sasser-Coen J. Memories of menarche: older women remember their first period. J Aging Stud. 1996;10(2):83-101. doi: 10.1016/S0890-4065(96)90007-9. PubMed PMID: 61475704; 9705889.
69. Lee S. Health and sickness: the meaning of menstruation and premenstrual syndrome in women's lives. Sex Roles. 2002;46(1-2):25-35. doi: 10.1023/A:1016033517659. PubMed PMID: 60084519; 200220521.
70. Li AD, Bellis EK, Girling JE, Jayasinghe YL, Grover SR, Marino JL, et al. Unmet needs and experiences of adolescent girls with heavy menstrual bleeding and dysmenorrhea: a qualitative study. J Pediatr Adol Gynec. 2020;33(3):278-84. doi: 10.1016/j.jpag.2019.11.007. PubMed PMID: 2004759150.
71. Lowik AJ. “Just because I don't bleed, doesn't mean I don't go through it”: Expanding knowledge on trans and nonbinary menstruators. Int J Trans Health. 2020. doi: 10.1080/15532739.2020.1819507.
72. Marshall J. An exploration of women's concerns about heavy menstrual blood loss and their expectations regarding treatment. J Reprod Infant Psyc. 1998;16(4):259-76. doi: 10.1080/02646839808404574. PubMed PMID: 57676951; 133263.
73. Marshall AL, Dasari H, Warner ND, Grill DE, Nichols WL, Pruthi RK. Self-reported reproductive health experiences in women with von Willebrand disease: a qualitative interview-based study. J Obstet Gynaecol. 2019;39(2):288-90. doi: 10.1080/01443615.2018.1472223.
74. Mason L, Cunningham C. An exploration of issues around menstruation for women with Down syndrome and their carers. J Appl Res Intellect. 2008;21(3):257-67. doi: 10.1111/j.1468-3148.2007.00406.x.
75. Matias-Gonzalez Y, Sanchez-Galarza AN, Flores-Caldera I, Rivera-Segarra E. "Es que tu eres una changa": stigma experiences among Latina women living with endometriosis. J Psychosom Obst Gyn. 2020. doi: 10.1080/0167482X.2020.1822807. PubMed PMID: 2006773151.
76. McKechnie R. The identification of menstrual change: working with biographies of reproduction. J Anthropol Soc Oxford. 2000;31(1):45-65. PubMed PMID: 60548183; 200500639.
77. Moas sG. The Value of Menstruation: Positive Meanings of the Female Lived-Body Experience [thesis]: Bar-Ilan University (Israel); 2010.
78. Murray S. “Being unwell”: menstruation in early Twentieth-Century Australia. In: Long J, Gothard J, Brash H, editors. Forging identities : bodies, gender and feminist history. Nedlands, Australia: University of Western Australia Press; 1997.
79. Murray S. 'Keeping their secret safe': menstrual etiquette in Australia, 1900-1960. Hecate. 1998;24(1):63-80.
80. Murray SM. A history of menstruation in Australia, 1900-1960 [thesis]: University of Western Australia (Australia); 1996.
81. Newton VL. Status passage, stigma and menstrual management: ‘starting’ and ‘being on’. 2012;10(4):392-407. doi: 10.1057/sth.2012.13.
82. Newton VL. Everyday Discourses of Menstruation. Basingstoke, UK: Palgrave Macmillan; 2016.
83. O’Flynn N, Britten N. Menorrhagia in general practice — disease or illness. Soc Sci Med. 2000;50(5):651-61. doi: 10.1016/S0277-9536(99)00318-4.
84. O'Flynn N. Menstrual symptoms: the importance of social factors in women's experiences. Brit J Gen Pract. 2006;56(533):950-7.

85. Oinas E. Young women's perspectives on public health knowledge and adolescent bodies. Scand J Public Healt. 1999;27(4):267-72. doi: 10.1177/14034948990270041101.
86. Owen L. Innovations in Menstrual Organization: Redistributing Boundaries, Capitals, and Labour 2020.
87. Pafford L. The culture and practices of military women relating to hygiene in the field environment [thesis]: Texas Woman's University (USA); 2007.
88. Pascoe C. Silence and the history of menstruation. Oral Hist Ass Aus J. 2007;(29):28-33.
89. Pascoe C. A 'Discreet Dance': technologies of menstrual management in Australian public toilets during the twentieth century. Women Hist Rev. 2015;24(2):234-51. doi: 10.1080/09612025.2014.948274.
90. Patterson ET, Hale ES. Making sure: integrating menstrual care practices into activities of daily living. Adv Nurs Sci. 1985;7(3):18-31. doi: 10.1097/00012272-198504000-00004. PubMed PMID: 00012272-198504000-00004.
91. Prileszky G. Medical management of heavy menstrual bleeding: understanding women's experiences [thesis]: The University of Nottingham (United Kingdom); 2013.
92. Raynor G. Invisible menstruators: transgender and genderqueer individuals' perceptions and experiences with menstruation: Texas State University; 2020.
93. Rodgers J. Pain, shame, blood, and doctors: How women with learning difficulties experience menstruation. Women Stud Int Forum. 2001;24(5):523-39. doi: 10.1016/S0277-5395(01)00195-9.
94. Rubinsky V, Gunning JN, Cooke-Jackson A. "I thought I was dying:" (un)supportive communication surrounding early menstruation experiences. Health Commun. 2020;35(2):242-52. doi: 10.1080/10410236.2018.1548337. PubMed PMID: 140918502.
95. Rydström K. Trans Menstruators Matter: A Qualitative Research Exploring Swedish Trans Experiences with Menstruation: University of Oviedo; 2018.
96. Santer M. Heavy menstrual bleeding: who suffers, who consults and why? [thesis]: The University of Edinburgh (United Kingdom); 2005.
97. Santer M, Wyke S, Warner P. What aspects of periods are most bothersome for women reporting heavy menstrual bleeding? Community survey and qualitative study. BMC Women's Health. 2007;7 (no pagination)(8). doi: 10.1186/1472-6874-7-8.
98. Santer M, Wyke S, Warner P. Women's management of menstrual symptoms: findings from a postal survey and qualitative interviews. Soc Sci Med. 2008;66(2):276-88. doi: 10.1016/j.socscimed.2007.08.018. PubMed PMID: 61698596; 200814294.
99. Sasser-Coen JR. The point of confluence: A qualitative study of the life-span developmental importance of menarche in the bodily histories of older women [thesis]: Oregon State University (USA); 1997.
100. Scott KD, Hintz EA, Harris TM. "Having pain is normal": how talk about chronic pelvic and genital pain reflects messages from menarche. Health Commun. 2020:1-11. doi: 10.1080/10410236.2020.1837464.
101. Sebert Kuhlmann A, Peters Bergquist E, Danjoint D, Wall LL. Unmet menstrual hygiene needs among low-income women. Obstet Gynecol. 2019;133(2):238-44. doi: 10.1097/AOG.0000000000003060.
102. Secor-Turner M, Andrea Huseth-Zosel, Rachel Ostlund. Menstruation experiences of middle and high school students in the Midwest: a pilot study. J Sch Nurs. 2020. doi: 10.1177/1059840520974234.
103. Seear K. The etiquette of endometriosis: Stigmatisation, menstrual concealment and the diagnostic delay. Soc Sci Med. 2009;69(8):1220-7. doi: 10.1016/j.socscimed.2009.07.023.
104. Segal AM. The experience of having endometriosis [thesis]: California School of Professional Psychology - Fresno (USA); 1998.
105. Skultans V. The symbolic significance of menstruation and the menopause. Man. 1970;5(4):639-51. doi: 10.2307/2799108. PubMed PMID: 60901154; 76H9505.
106. Skultans V. Menstrual symbolism in South Wales. In: Buckley T, Gottlieb A, editors. Blood Magic: The Anthropology of Menstruation. Berkeley, USA: University of California Press; 1988. p. 137-60.
107. Sommer M, Gruer C, Smith RC, Maroko A, Kim H. Menstruation and homelessness: Challenges faced living in shelters and on the street in New York City. Health Place. 2020;66:102431. doi: 10.1016/j.healthplace.2020.102431.

108. Statham G. Understanding the effects of the menstrual cycle on training and performance in elite athletes: A preliminary study. Prog Brain Res. 2020;253:25-58. doi: 10.1016/bs.pbr.2020.05.028. PubMed PMID: 2007139663.
109. Steward R, Crane L, Mairi Roy E, Remington A, Pellicano EJJoA, Disorders D. "Life is much more difficult to manage during periods": autistic experiences of menstruation. J Autism Dev Disord. 2018;48(12):4287-92. doi: 10.1007/s10803-018-3664-0.
110. Teitelman AM. Adolescent girls' perspectives of family interactions related to menarche and sexual health. Qual Health Res. 2004;14(9):1292-308. doi: 10.1177/1049732304268794. PubMed PMID: 57171658; 319128.
111. Thuren BM. Opening doors and getting rid of shame - experiences of 1st menstruation of Valencia, Spain. Women Stud Int Forum. 1994;17(2-3):217-28. doi: 10.1016/0277-5395(94)90027-2. PubMed PMID: WOS:A1994NM91600010.
112. Tingle C, Vora S. Break the Barriers: Girls' experiences of menstruation in the UK. London, UK: 2018.
113. Tolson D, Fleming V, Schartau E. Coping with menstruation: understanding the needs of women with Parkinson's disease. J Adv Nurs. 2002;40(5):513-21. doi: 10.1046/j.1365-2648.2002.02408.x. PubMed PMID: 57200324; 287577.
114. Trego LL. Military women's menstrual experiences and interest in menstrual suppression during deployment. JOGNN. 2007;36(4):342-7. doi: 10.1111/j.1552-6909.2007.00166.x. PubMed PMID: 106151338.
115. Uskul AK. Women's menarche stories from a multicultural sample. Soc Sci Med. 2004;59(4):667-79. doi: 10.1016/j.socscimed.2003.11.031. PubMed PMID: 57043172; 300520.
116. Vora S. Tackling Period Poverty Report 2017. Bristol, UK: No More Taboo, 2017.
117. Vora S. The Realities of Period Poverty: How homelessness shapes women's lived experience of menstruation. In: Bobel C, Winkler IT, Fahs B, Hasson KA, Kissling EA, Roberts T-A, editors. The Palgrave Handbook of Critical Menstruation Studies: Palgrave Macmillan; 2020.
118. Whisnant L, Zegans L. A study of attitudes toward menarche in white middle-class American adolescent girls. Am J Psychiat. 1975;132(8):809-14. doi: 10.1176/ajp.132.8.809. PubMed PMID: 5530794.
119. Wigmore-Sykes M, Ferris M, Singh S. Contemporary beliefs surrounding the menarche: a pilot study of adolescent girls at a school in middle England. Educ Prim Care. 2020. doi: 10.1080/14739879.2020.1836678.
120. Wood JM, Koch PB, Mansfield PK. Is my period normal? How college-aged women determine the normality or abnormality of their menstrual cycles. Women Health. 2007;46(1):41-56. doi: 10.1300/J013v46n01_04. PubMed PMID: 18032174.
121. Wootton S, Morison T. Menstrual management and the negotiation of failed femininities: a discursive study among low-income young women in Aotearoa (New Zealand). Women Reprod Health. 2020;7(2):87-106. doi: 10.1080/23293691.2020.1740485.
