# Supplementary material for: Experiences of menstruation in high income countries: A systematic review, qualitative evidence synthesis and comparison to low- and middle-income countries
Source: PLoS One. 2021 Jul 21;16(7):e0255001. doi: 10.1371/journal.pone.0255001 (PMC8294489; doi:10.1371/journal.pone.0255001)
Supplement: Attachment — Submitted filename: Response to Reviewers.pdf [file pone.0255001.s005.pdf]

Response to Reviewers

PONE-D-21-08777

Experiences of menstruation in high income countries: a systematic review, qualitative evidence synthesis and comparison to low- and middle-income countries

**Editor**	
Please ensure that your manuscript meets PLOS ONE's style requirements, including those for file naming.	The manuscript has been reformatted to meet PLOS ONE's style requirements. For ease of reading, we have not tracked these formatting changes. All submission files abide by PLOS ONE naming conventions.
Please revise your PRISMA flow chart to ensure that you have included the reasons that full text articles were removed (listing how many were excluded for each reason).	Figure 1 (PRISMA flow chart) has been revised.
Please ensure that you have stated all inclusion and exclusion criteria used to select manuscripts for inclusion.	All inclusion and exclusion criteria have been stated.
We note that you use the word "meta-synthesis" in your title. As some readers may find this misleading as it is similar to "meta-analysis" please revise your title appropriately.	We have revised to "qualitative evidence synthesis," as the dominant terminology now being used for this type of review.
In your Data Availability statement, you have not specified where the minimal data set underlying the results described in your manuscript can be found. PLOS defines a study's minimal data set as the underlying data used to reach the conclusions drawn in the manuscript and any additional data required to replicate the reported study findings in their entirety. All PLOS journals require that the minimal data set be made fully available.	We have amended our Data Availability statement to:<br>This manuscript made use of secondary data in the form of publications reporting on menstrual experiences in high income countries. Table 2 and the Reference list provide the details of all publications included in this systematic review.
Please include captions for your Supporting Information files at the end of your manuscript, and update any in-text citations to match accordingly.	Captions for Supporting Information have been formatted to PLOS ONE style and included at the end of the manuscript.

Please review your reference list to ensure that it is complete and correct.	The reference list is complete and correct and conforms to PLOS ONE's style guidelines.
a) ensure consistency in spelling meta-synthesis (sometimes metasynthesis)	We have revised this to 'qualitative evidence synthesis' consistent with the revisions to the title.
b) minor edits upset, like (line 341); line 716 - However...	We have reviewed for typos and made corrections as necessary.
c) please check and confirm individuals acknowledged by name are happy with this	The individuals named in the acknowledgements have agreed to their inclusion in the manuscript.
**Reviewer**	
Abstract: The background provided is relevant, but it seems like a final sentence laying out more explicitly the aim of the present study would be helpful for readers before launching into the methods.	We have added the following sentence to the Background section of the Abstract: "To inform the growing policy attention to support people who menstruate, here we review and synthesise the existing research."
Data analysis: Authors discuss "coding studies", but it is not clear exactly what data were coded. It would be helpful to have a quick mention of the process of extracting data from the published studies that was then used in the meta-synthesis. (e.g. Did the authors code any/all information that appeared in the "results" sections of published papers? Or did they also include information from "discussion" sections? Did they analyze author interpretations or only direct quotations from original studies, etc.)	The coding method is now fully explained; "DJB coded the study results, quotations from those who menstruate and author interpretations in studies of high and medium trustworthiness in NVivo 12"
Data analysis: In step 4, two other authors coded 30% of the studies for validation purposes "without having viewed the coding template". What type of coding were these authors conducting? Their approach is unclear—were they doing only inductive coding or did they also use the same framework approach as the first author (just without viewing what new codes were added by the first author)?	The manuscript text has been amended to "Using the same approach as DJB, they deductively coded studies against the framework of themes from the review of studies in LMICs and inductively identified new codes independently, that is, without having reviewed DJB's coding template.. DJB reviewed co-author coding and it was consistent with the coding template"

Data analysis: In this section, it is unclear what "third-order constructs" is referring to in the analysis. Which are considered first and second-order constructs? This terminology does not come up again in the findings.	We agree this may not be familiar terminology to readers, and is unnecessary. We have revised the sentences to read:<br>"We used a combination of line-by-line coding and thematic network mapping to identify overarching themes and develop our final synthesis"<br>"The final model and themes are contrasted against findings from the review of LMIC studies in the Discussion, facilitated by the process of coding against these themes during our analysis."
Table 2: The authors might consider changing the column "population size" to "number of participants" to more accurately describe the contents of that column	This has been amended.
Table 2: Have the data collection methods of "focus group discussions" been combined/collapsed into the category of "group interviews"? Some disciplines distinguish between a group interview and focus group discussion (they are not considered the same method). In the table contents, it appears only the term "group interview" in used, yet in the footnote 2, the term "focus group" is employed. The authors might want to provide clarity around this.	Focus group discussions and group interviews were collapsed into the category of 'group interviews'. Table 2, Footnote 2, has been amended to reflect this, as well as the description within the text, "23 used group interviews (including focus group discussions)"
Table 2: In the column "author stated analytical method," some of the things listed are analysis methods but some are epistemological perspectives and/or theories or frameworks (social constructionism & symbolic interactionism) that guide methods but are not analysis "methods" in themselves.	This column has been renamed, 'Author stated analytical method and/or epistemological perspective'. Also, we have replaced 'unclear' in this column with 'not stated', as all of the methods/perspectives listed here are those stated by the authors of the original study, not as interpreted by the authors of this manuscript.
Results: At the end of line 338, I think a word is missing after "regards". Should this be "with regards to menarche"? (same issue in line 486, 554)	This has been amended.

Results: In line 366, it is unclear whether the "media rhetoric that stigma around menstruation in HICs is decreasing" was a finding from the studies in the review. This may be more appropriate to discuss in the discussion section rather than the findings if not.	The reference to media rhetoric has been removed.
Discussion: Information in lines 791-797 appears to be new results that were not presented earlier in the findings section.	The findings below are discussed in lines 791-797 of the original submission (lines 802-810 of the revised submission). We have added details of these fundings to the Results section where necessary.<br><br>We provide below the reference to each finding in the Results section (line number in brackets), with changes tracked in the accompanying document:<br><br>Individual menstrual factors such as pain, fatigue and gastrological and neurological symptoms were commonly associated with negative experiences (lines 562-563, 598), and led to increased mental burden (lines 662), as well as detrimental impacts on participation (lines 604, 705) and relationships (line 603).<br><br>Participants who experienced irregular periods suffered particularly high mental burden from the constant need to be 'prepared' in case menstrual bleeding began unexpectedly (line lines 662-664). Those with menstrual disorders expended significant energy hiding their symptoms from employers and/or feeling guilty about letting colleagues and family members down (lines 371-373, 689-6902). However, where healthcare workers were supportive and pain management effective, some participants did feel relief and reduced mental burden, and saw an improvement in their participation and relationships (lines 387-390).
